# Bridging the Gap Between hiPSC-CMs Cardiotoxicity Assessment and Clinical LVEF Decline Risk: A Case Study of 21 Tyrosine Kinase Inhibitors

**DOI:** 10.3390/ph18040450

**Published:** 2025-03-23

**Authors:** Zhijie Wan, Chenyu Wang, Shizheng Luo, Jinwei Zhu, Hua He, Kun Hao

**Affiliations:** 1 State Key Laboratory of Natural Medicine, Jiangsu Province Key Laboratory of Drug Metabolism and Pharmacokinetics, China Pharmaceutical University, Nanjing 210009, China; 2 Center of Drug Metabolism and Pharmacokinetics, China Pharmaceutical University, Nanjing 210009, China

**Keywords:** QSP-PK-PD model, hiPSC-CMs, TKIs, cardiotoxicity, in vitro to in vivo extrapolation (IVIVE)

## Abstract

**Objectives**: There is growing concern over tyrosine kinase inhibitor (TKI)-induced cardiotoxicity, particularly regarding left ventricular dysfunction and heart failure in clinical treatment. These adverse effects often lead to treatment discontinuation, severely impacting patient outcomes. Therefore, there is an urgent need for more precise risk assessment methods. This study aimed to assess the cardiotoxicity of TKIs, refine in vitro to in vivo extrapolation (IVIVE) methodologies to improve predictive accuracy, and identify critical in vitro parameters for assessment. **Methods**: By leveraging high-throughput cardiotoxicity screening with human induced pluripotent stem cell-derived cardiomyocytes (hiPSC-CMs), a mechanism-based toxicodynamic (TD) model for TKIs was constructed. A QSP-PK-TD model was developed by integrating pharmacokinetic (PK) and quantitative systems pharmacology (QSP) models. This model incorporates critical drug exposure factors, such as plasma protein binding, tissue–plasma partitioning, and drug distribution heterogeneity to enhance extrapolation accuracy. **Results**: The QSP-PK-TD model validated the reliability of IVIVE and identified the area under the curve of drug effects on mitochondrial membrane potential (AEMMP) and cardiomyocyte contractility (AEAAC) as key in vitro parameters for assessing TKI-induced cardiotoxicity. Incorporating critical drug exposure factors obviously improved qualitative and quantitative extrapolation accuracy. **Conclusions**: This study established a framework for predicting in vivo cardiotoxicity from in vitro parameters, enabling efficient translation of preclinical data into clinical risk assessment. These findings provide valuable insights for drug development and regulatory decision-making, offering a powerful tool for evaluating TKI-induced cardiotoxicity.

## 1. Introduction

The emergence of tyrosine kinase inhibitors (TKIs) has greatly improved progression-free and overall survival of cancer patients. However, this benefit may be undermined by drug-related cardiotoxicity [1]. Left ventricular systolic dysfunction and heart failure are common issues that can lead to therapy discontinuation [2,3]. Cardiac dysfunction caused by TKIs might be concealed by compensatory mechanisms, like an increase in cardiac output through the Frank–Starling mechanism [4,5], particularly in patients without pre-existing cardiovascular conditions. Consequently, these issues are often identified later in drug development or post-marketing, necessitating innovative methods to assess drug-induced cardiac dysfunction in cancer patients.

TKI-induced left ventricular systolic dysfunction by disrupting cardiomyocyte viability and contractility [6]. They may generate reactive oxygen species (ROS), leading to cardiomyocyte apoptosis [7]. TKIs also inhibit growth factor and cytokine signaling, impairing cardiomyocyte survival during stress and suppressing myocardial contraction through various signaling pathways such as the unfolded protein response, protein kinase B, and extracellular signal-regulated kinase signaling pathways [8,9,10]. Additionally, TKIs disrupt cardiac mitochondrial metabolism, reducing energy production and myocardial contraction [8].

The emergence of human-induced pluripotent stem cell-derived cardiomyocytes (hiPSC-CMs) provides a platform for high-throughput evaluation of drug-induced cardiotoxicity, assessing cell viability, energy metabolism, and contractility [7,11,12]. Sharma et al. [12,13] proposed a cardiac safety index using hiPSC-CMs-based tests to identify potential cardiotoxic agents, linking in vitro findings with clinical risk prediction. Accurately predicting clinical cardiotoxicity risk for antineoplastic agents is crucial for balancing the risk–benefit ratio. Thus, a quantitative in vitro to in vivo extrapolation (QIVIVE) approach is vital for informed decision-making in drug development and therapy for high-risk patients.

In toxicology predictions, maximal plasma drug concentration (C_max_) is often used as the exposure metric for in vitro–in vivo extrapolation (IVIVE). However, drug toxicity is mainly driven by target tissue exposure, which can differ from plasma concentrations. The free drug theory states that unbound drug concentrations in plasma and tissue interstitial fluid are equal at steady state [14]. However, factors such as lipoidal permeability, pH differences, transporter presence, and drug–target residence time can significantly affect target drug exposure [14,15,16,17]. Additionally, heterogeneous drug distribution within tissues may influence drug-induced cardiotoxicity. For example, hiPSC-CMs in three-dimensional (3D) models better mimic in vivo conditions than traditional two-dimensional (2D) cultures [18]. To improve QIVIVE precision in predicting drug-induced cardiotoxicity, factors affecting target drug exposure must be considered.

In our previous study, we used hiPSC-CMs and IVIVE to assess drug-induced cardiotoxicity [19], evaluating drug effects on cell viability, energy metabolism, and contractility. The results were integrated into the IVIVE platform, which accurately predicted the cardiotoxic effects of doxorubicin and trastuzumab. This study aimed to utilize the IVIVE platform to assess the cardiotoxicity of 21 TKIs and identify key parameters for evaluating cardiotoxicity through in vitro experiments. Furthermore, the study sought to refine the QIVIVE model by incorporating adjustment factors such as plasma protein binding, tissue partitioning, and heterogeneous drug distribution. These refinements were expected to enhance the accuracy of predicting TKI-induced cardiotoxicity and improve the translation of in vitro findings to clinical applications.

## 2. Results

### 2.1. TD Model of TKIs

**Figure 1:**
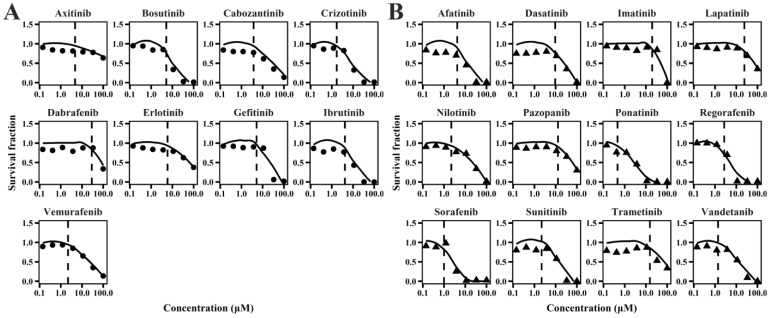
Individual plots of TKIs on survival fraction of hiPSC-CMs for TKIs with no cardiotoxicity (**A**) and TKIs with cardiotoxicity (**B**).

**Figure 2:**
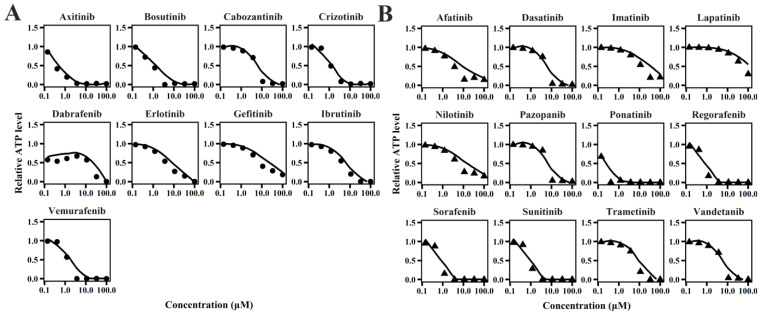
Individual plots of TKIs on relative ATP level of hiPSC-CMs for TKIs with no cardiotoxicity (**A**) and TKIs with cardiotoxicity (**B**).

**Figure 3:**
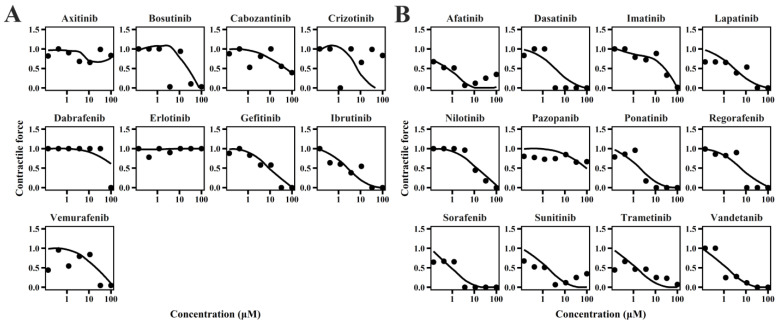
Individual plots of TKIs on contractile force of hiPSC-CMs for TKIs with no cardiotoxicity (**A**) and TKIs with cardiotoxicity (**B**).

The cellular TD models effectively describe changes in cell viability, ATP levels, and contraction force of hiPSC-CMs (Figure 1, Figure 2 and Figure 3) following TKI treatment [12]. The estimated model parameters are summarized in Table 1. A toxic effect on cell viability was detected at concentrations as low as 0.1 μM for doxorubicin, a cytotoxic agent [19]. The minimal concentration at which TKIs exhibited toxic effects on viability was approximately 1 μM, suggesting distinct mechanisms of cardiotoxicity between cytotoxic and noncytotoxic agents. To account for this difference, a threshold concentration was introduced for TKIs’ negative impact on cell viability at lower concentrations (Table S1). There is no significant difference in the estimated threshold between cardiotoxic and noncardiotoxic TKIs (Figure 1). All TKIs showed a concentration-dependent reduction in ATP levels after 72 h (Figure 2), with estimated half-maximal effective concentration (EC_50MMP_) ranging from 5.54 to 50.15 μM for noncardiotoxic TKIs and 2.54 to 69.6 μM for cardiotoxic TKIs. The effects on cellular contractility after 72 h are presented in Figure 3. Concerning noncardiotoxic TKIs, such as axitinib, bosutinib, cabozantinib, dabrafenib, and erlotinib, the drug effect on cellular contractility (EEC) may be contributed by the reduced ATP generation as they performed no effect on ATP_50_ref_. Pazopanib was the only TKI with cardiotoxicity that performed no effect on ATP_50_ref_, suggesting the remarkable decline in ATP generation may also compromise cardiomyocyte contractility.

### 2.2. Quantitative Evaluation of TKI-Induced Cardiac Dysfunction

**Figure 4:**
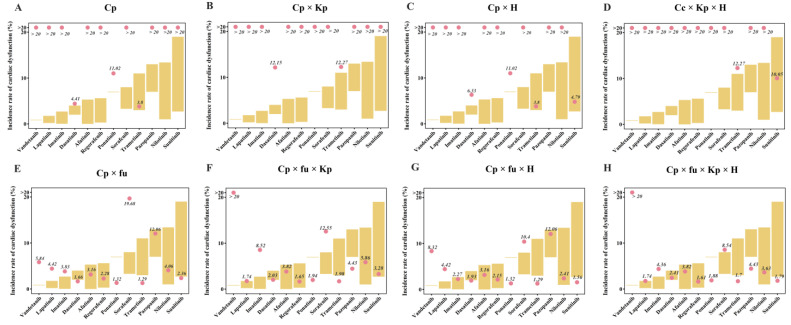
Quantitative assessments based on the QSP-PK-TD model. The different subfigures represent the correction of drug concentration using different factors. Cp, plasma drug concentration. Fu, plasma protein binding. Kp, tissue–plasma partition coefficient. H, heterogeneous distribution.

TKI-induced cardiac dysfunction was quantified using the previously developed IVIVE method [19]. This approach allowed for the simulation of systolic dysfunction incidence in patients with varying cardiovascular conditions. For TKIs with reported incidence of cardiac dysfunction, we conducted a virtual clinical trial to predict the incidence rates of this adverse effect. The quantitative analysis demonstrated that refining the effective drug concentration by incorporating the parameters fu, K_p_, and H improved prediction accuracy. Predicted incidence rates for most TKIs were found to fall within a twofold range of the observed clinical rates (Figure 4). Furthermore, the incidence rates for hypertensive and dilated cardiomyopathy patients were approximately 3.27-fold and 3.53-fold higher, respectively, compared to healthy individuals (Figure S1), which is in close agreement with the clinically reported 3.63-fold increase [20].

Initial estimates based solely on plasma drug concentration significantly overestimated the incidence rates of cardiac dysfunction, emphasizing the importance of considering unbound plasma drug concentration for accurate predictions. As shown in Figure 4, excluding fu led to overpredictions, while including it improved accuracy. Although adding K_p_ and/or H resulted in modest improvements, combining all three factors (fu, K_p_, and H) yielded the best predicted incidence rates. Vandetanib was an exception, with a clinical incidence rate of 0.9%. The model predicted 5.84% using plasma-free drug concentration, which increased to 8.32% with H and over 20% with K_p_, indicating that the overestimated K_p_ value was the main contributor to the overprediction of vandetanib’s incidence rate of cardiac dysfunction.

### 2.3. Results of Sensitivity Analysis

**Figure 5:**
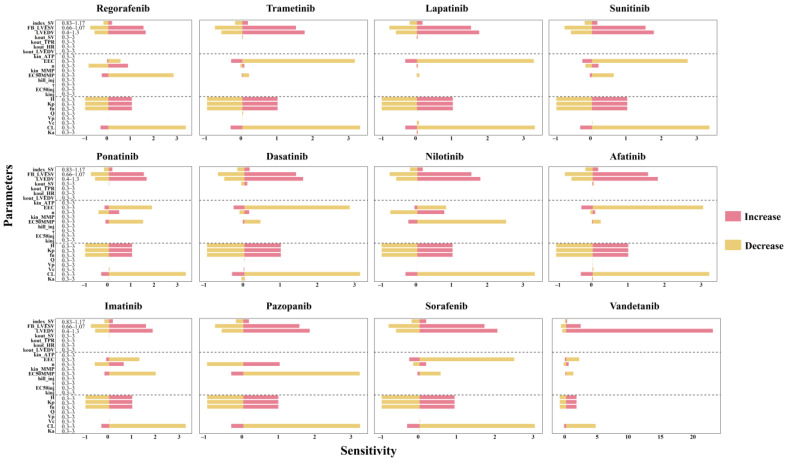
Sensitivity analysis of the QSP-PK-TD model. The systematic parameters in QSP model were from a previous study [21]. HR, heart rate. TPR, total peripheral resistance. SV, stroke volume. LVEDV, left ventricular end-diastolic volume. LVESV, left ventricular end-systolic volume. index_SV, LVEDV correction coefficient of SV. FB_LVESV, feedback constant of LVESV on dissipation of LVEDV. kin_HR, kin_TPR, kin_LVEDV, and kin_SV are the zero-order production rate constants, while kout_HR, kout_TPR, kout_LVEDV, and kout_SV are the first-order dissipation rate constants for HR, TPR, LVEDV, and SV, respectively.

The sensitivity analysis of the QSP, TD, and PK model parameters is presented in Figure 5. Clearance (CL) is identified as the most sensitive parameter, with a sensitivity index of approximately 3.2 when CL decreases. Among the systematic parameters within their physiological ranges (as outlined in Table in Section 4.3), LVEDV and FB_LVESV were identified as sensitive systemic parameters. The TD parameters EEC and EC_50MMP_, and the PK parameters fu, K_p_, and H were also identified to be sensitive. When the values of these parameters increase by threefold, the sensitivity indices for fu, K_p_, and H are all approximately 1. Additionally, when EC_50MMP_ is smaller than that of EEC, the EC_50MMP_ of TKIs is more sensitive. TKIs with a smaller initial value of EEC exhibit greater sensitivity.

To further elucidate the relationship between EEC and EC_50MMP_ and their corresponding sensitivity indices, a Pearson correlation analysis was conducted (Figure S2). The results indicated that the sensitivity index is correlated with EEC but not with EC_50MMP_. Lower values of EEC are associated with higher sensitivity, suggesting that drug-induced cardiomyopathy is more likely to result from functional impairments in the myocardium rather than disruptions in energy metabolism.

### 2.4. Qualitative Assessment of TKI-Induced Cardiac Dysfunction

Given the limited effects of TKIs on cell viability, their impact on bioenergy production and cellular sensitivity to bioenergy is crucial for understanding TKI-induced cardiotoxicity. We simulated the effects of toxic TKIs on MMP and AAC, revealing that both EMMP and EAAC reach steady-state levels as dosing time increased (Figure S3). To distinguish between toxic and nontoxic TKIs, we introduced two PK-TD parameters—AEAAC (area under the effect curve of EAAC) and AEMMP (area under the effect curve of EMMP). These parameters integrate drug exposure and TD characteristics, quantifying a drug’s impact on bioenergy production and cellular sensitivity to bioenergy and serving as key indicators of drug-induced cardiotoxicity.

**Figure 6:**
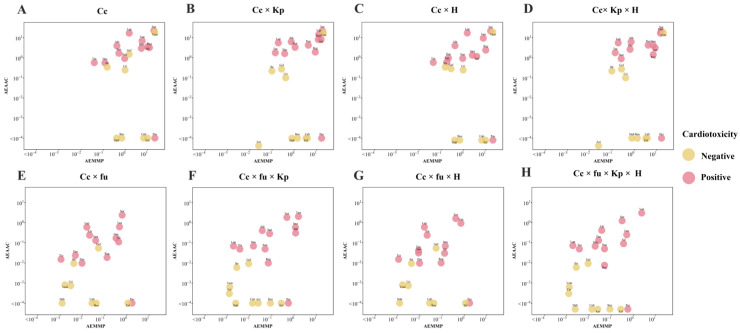
Qualitative assessments based on the QSP-PK-TD model. The different subfigures represent the correction of drug concentration using different factors. Cp, plasma drug concentration. Fu, plasma protein binding. Kp, tissue–plasma partition coefficient. H, heterogeneous distribution.

When plasma drug concentration was used to calculate PK-TD parameters, AEAAC and AEMMP had limited ability to distinguish cardiotoxic from noncardiotoxic TKIs (Figure 6). However, incorporating additional factors, such as the tissue–plasma partition coefficient, plasma-free fraction, and a heterogeneity factor, improved the accuracy of AEAAC and AEMMP to identify cardiotoxic drugs. Among these factors, K_p_ demonstrated superior discriminative ability compared to plasma-free fraction and the heterogeneity factor, with vemurafenib being the only TKI misclassified. The combination of fu, K_p_, and H successfully differentiated cardiotoxic from noncardiotoxic TKIs. As illustrated in Figure 6, cardiotoxic TKIs exhibited higher AEAAC and AEMMP values compared to noncardiotoxic TKIs, highlighting the predictive value of these parameters for assessing the risk of cardiotoxicity.

### 2.5. The Combined Effects of PK-TD Parameters on AEMMP and AEAAC

**Figure 7:**
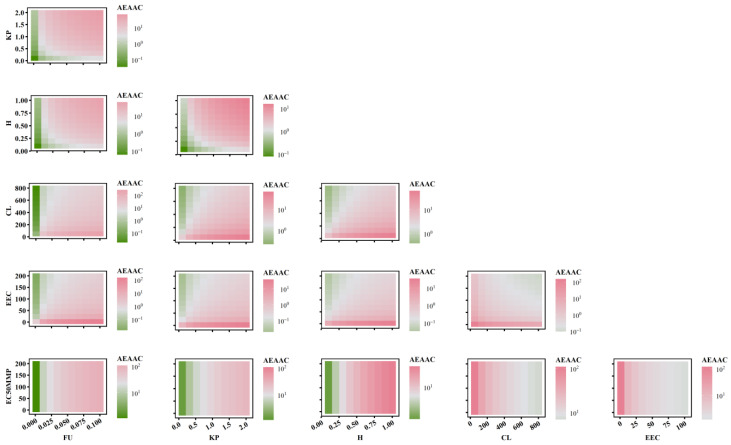
The combined effects of PK-TD parameters on AEAAC.

**Figure 8:**
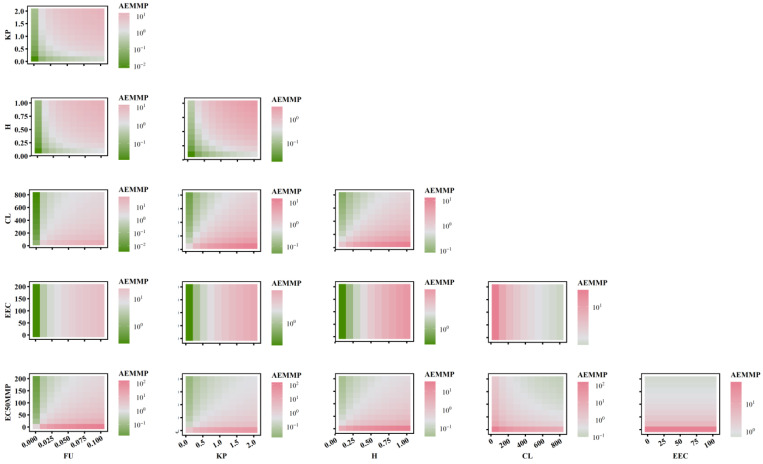
The combined effects of PK-TD parameters on AEMMP.

To further elucidate the relationship between AEAAC, AEMMP, and the sensitivity of PK-TD parameters, the combined effects of two factors on AEMMP and AEAAC are illustrated in Figure 7 and Figure 8. The results demonstrate that, under the same parameter values, PK-TD parameters have a greater impact on AEAAC than on AEMMP, as evidenced by their respective numerical values. This suggests that AEAAC is more sensitive to changes in PK-TD factors compared to AEMMP. Given that cardiotoxic TKIs exhibit higher AEAAC and AEMMP values, it is evident that smaller AEMMP and AEAAC values correlate with lower cardiotoxicity. Specifically, lower values of fu (free fraction of the drug), K_p_ (plasma-to-tissue distribution coefficient), and H (drug heterogeneous distribution), combined with higher values of CL (clearance rate), EEC, and EC_50MMP_, are all associated with significantly reduced AEMMP and AEAAC values. Furthermore, TKIs with low K_p_ values but high CL values are associated with a reduced cardiotoxicity risk. That indicates that drugs with lower tissue distribution (small K_p_) and higher clearance (larger CL) are less likely to accumulate in the heart, lowering the potential for cardiac toxicity.

When analyzing the impact of EC_50MMP_ (drug effect on energy metabolism) relative to other PK parameters on AEAAC, the study found that PK parameters predominantly influence AEAAC. Specifically, when K_p_ is less than 1, AEAAC values consistently remain below 10, regardless of variations in EC_50MMP_. Similarly, when examining the effect of EEC (myocardial contractility) in relation to other PK parameters on AEMMP, PK parameters again exhibited a dominant influence. For instance, when K_p_ is less than 1, AEMMP values consistently remain below 1, irrespective of changes in EEC. These findings suggest that PK factors (e.g., K_p_, CL) exert a significantly stronger influence on AEAAC and AEMMP than TD factors (e.g., EC_50MMP_ and EEC). These findings highlight the critical role of PK parameters in governing drug distribution, metabolism, and clearance, which directly modulate the extent of drug accumulation in the heart and serve as a primary driver in predicting cardiac toxicity risk. While TD factors such as the drug’s effects on energy metabolism and myocardial contractility contribute to cardiotoxicity, their influence is secondary to the dominant impact exerted by PK factors. This underscores the importance in prioritizing PK optimization in the assessment and mitigation of cardiac toxicity risks.

## 3. Discussion

In recent decades, cardiotoxicity assessment has primarily targeted arrhythmogenic risk, particularly due to the life-threatening potential of drug-induced torsade de pointes. The adoption of preclinical screening methods, such as the Comprehensive in vitro Proarrhythmic Assay, has significantly reduced the incidence of drug-associated QT prolongation [21,22]. However, a new concern has arisen as antitumor treatments are occasionally halted or discontinued due to antineoplastic drugs causing left ventricular dysfunction and heart failure. Utilizing high-throughput hiPSC-CMs, this model assessed drug effects through assays for cell viability, contractility, and mitochondrial function. To quantify the relationship between contractility and LVEF, the QSP model was integrated to describe the interactions among contractility, preload, and afterload [23]. In this study, a QSP-PK-TD model was developed to translate drug effects from cardiomyocytes to humans, enabling the prediction of cardiac dysfunction incidence.

Drug-induced cardiac dysfunction is driven by a complex interplay of mechanisms, including apoptosis and mitochondrial dysfunction in cardiomyocytes [7,24,25]. TKI-induced cardiac dysfunction is primarily characterized by functional impairments, such as reduced myocardial systolic and diastolic performance, as well as disturbances in myocardial bioenergy production [26,27]. Importantly, these impairments are not always associated with significant cardiomyocyte. Energy metabolism plays a crucial role in this process, particularly through the impact of the drug on mitochondrial function and ATP production. Insufficient myocardial energy supply exacerbates these functional deficits, further compromising cardiac performance [28,29]. Sensitivity analysis of the QSP-PK-TD model revealed that TD parameters representing cardiomyocyte structure and energy metabolism (EEC and EC_50MMP_) play important roles in TKI-induced cardiac dysfunction. This study suggests that TKI-induced cardiac dysfunction is primarily driven by functional impairments, with energy metabolism—especially mitochondrial function and ATP production—playing a key role in the TKI-induced cardiac dysfunction.

This “bottom-up” method allows the assessment of in vivo risk based on models built from in vitro data [30,31]. Since the EPA’s ToxCast research program was launched in 2007, an increasing number of studies have used in vitro high-throughput screening to test the toxicity of compounds [32,33]. The first phase of the ToxCast project was used to construct models aimed at determining the prediction of in vivo effects from in vitro data only. Computational chemistry, high-throughput screening, and various toxicogenomic techniques were used to predict potential toxicity. Traditional toxicological testing involves screening of compounds by in vivo and in vitro tests, but due to insufficient knowledge of the models or mechanisms, it is not possible to design suitable in vitro tests [34]. In this study, a mechanism-based QSP-PK-TD model was proposed to estimate the in vivo toxicity of TKIs through in vitro assessments. Using hiPSC-CMs assays, the effects of TKIs on, ATP, and AAC were evaluated. Notably, the cardiotoxic risk associated with TKIs was reduced when both AEMMP and AEAAC values from in vitro experiments were below 1. For a given drug, the incidence of cardiotoxicity could potentially be assessed based on the combination of its in vitro PD parameters and PK parameters, such as AEMMP and AEAAC.

This mechanism-based translational model enabled the estimation of clinical incidence rates of left ventricular dysfunction as a measure of drug-associated cardiotoxicity using previous in vivo–in vitro extrapolation methods [19]. Combining in vitro toxicity data with kinetic modeling enhances assessments of in vivo drug exposure [35]. One of these methods, quantitative in vitro to in vivo extrapolation, allows qualitative in vitro data to estimate drug exposure levels in target human tissues [36]. Previous studies have validated the QSP model in predicting cardiac dysfunction incidence rates with doxorubicin, where plasma drug concentration served as the driver of cardiotoxicity in the QSP-PBPK-TD model [19,23]. Yet, in vitro-based risk assessments face complexities beyond those of in vivo data applications, due to variations in clearance, protein binding, bioavailability, and other pharmacokinetic factors [32]. This highlights the ongoing need to refine translational models for improved accuracy in predicting drug-associated cardiac dysfunction. For instance, Luise et al. [37] found that QIVIVE improves predictive accuracy when protein binding is considered, underscoring the importance of accounting for differences in free drug concentration, tissue concentration, and distribution between in vitro and in vivo environments [38].

In translational modeling, free drug concentration in plasma often predicts drug efficacy [39]. Plasma drug unbound concentration would decrease available drugs for the generation of TKIs’ cardiotoxicity in vivo. That is because most tyrosine kinase inhibitors have high plasma protein binding. For example, sorafenib has a plasma protein binding rate of 99.5% [40] and regorafenib has a plasma protein binding rate higher than 95% [41]. The predicted incidence rates of cardiac dysfunction for all cardiotoxicity TKIs support this, as they decreased when drug exposure was corrected for plasma protein binding (Figure 4). These results underscore the importance of precisely determining protein binding in in vitro to in vivo translation.

Drug distribution influences not only efficacy but also toxicity [42,43]. Higher tissue–plasma partition coefficients could lead to drug accumulation in nontarget tissues, such as the heart, where elevated drug concentrations could induce toxic reactions [44,45]. Drug distribution varies based on drug properties (e.g., lipid solubility, molecular weight) and tissue characteristics (e.g., blood flow, membrane permeability, and plasma protein binding) [14,15,16,17,46,47]. Tissue-to-plasma ratio from preclinical determination or PBPK model-based prediction was often collected for in vitro to in vivo extrapolation [46,48]. As free drug concentration near therapeutic targets often determines efficacy [14], tissue accumulation alone may not contribute to cardiotoxicity prediction improvements, as the accumulated drug may bind to lipids or proteins without elevating active concentrations in cardiomyocytes [14]. That may account for the incidence of cardiotoxicity for most TKIs being overestimated. Accordingly, tissue accumulation may not be a critical factor for cardiotoxicity prediction accuracy.

Another major difference between the in vitro and in vivo systems lies in cardiomyocyte drug accessibility. In 2D culture, cardiomyocytes were uniformly exposed to drug identical concentrations. However, in 3D and cardiac tissues, drug concentrations exhibited a gradient distribution across different cell layers [49]. The determined IC_50_ is higher in 3D hiPSC-CMs, suggesting the reduced drug effect [12,17,50]. For example, Sang Lan et al. [19] introduced an accessibility parameter to represent drug distribution in interstitial fluid for in vitro to in vivo extrapolations. Cardiomyocytes within the tissue may thus experience lower drug concentrations, reducing drug effects. Accounting for heterogeneous drug distribution improved predictions, such as for sorafenib and imatinib.

When three key factors affecting drug exposure are incorporated, the QSP-PK-TD model not only improves the accuracy of the in vitro–in vivo translational, but also qualitatively distinguishes the cardiotoxicity of TKIs by PK-TD parameters. However, quantitative predictions for vandetanib and ponatinib were less accurate. This discrepancy may stem from vandetanib’s role as a vascular endothelial growth-factor receptor tyrosine kinase inhibitor (VEGFR-TKI), which can indirectly mitigate cardiovascular damage through effects on VEGFR signaling [51]. The QSP-PK-TD model did not account for this cardioprotective mechanism, potentially leading to an overestimation of cardiotoxicity. In contrast, ponatinib, associated with a significant risk of severe arrhythmia and QT interval prolongation at an incidence rate of 7.7% [52], was underpredicted by the model. This underestimation likely occurred because the QSP-PK-TD model focused on drug-induced cell fraction, energy metabolism, and myocardial contractility, without incorporating mechanisms specific to arrhythmogenic or QT-prolonging effects.

Additionally, sensitivity analysis revealed that a patient’s disease state has a substantial impact on the incidence of cardiac dysfunction. Systemic parameters had a greater influence on cardiotoxicity, and FB_LVESV and LVEDV were particularly impactful. Systemic parameters, especially FB_LVESV and LVEDV, greatly influence cardiotoxicity. When LVEDV reaches 145.8 mL, the heart may struggle to pump blood effectively, leading to increased blood volume at the end of diastole [53]. Consequently, stroke volume may not increase and could decline due to insufficient contractile force. FB_LVESV, which reflects different cardiac functional states, slows LVEDV elimination when increased, resulting in higher LVEDV and lower LVEF. For virtual clinical trials, parameters should be adjusted based on cardiac function status, and assessing LVEDV before administration while closely monitoring cardiac function is advised.

Compared to physiologically based pharmacokinetic (PBPK) models, the method proposed in this study offers significant advantages in the early stages of drug screening. PBPK models can integrate multiple physiological parameters, such as inter-organ blood flow dynamics, enzyme metabolic capacity, and transporter-mediated drug distribution, thus excelling in mechanism studies and personalized drug dosing optimization [54,55]. However, PBPK models involve many physiological variables, with high difficulty in parameter acquisition and complex modeling and calculation processes [56]. This limits its application in the early screening of new compounds, particularly during high-throughput screening. In contrast, the method proposed in this study, based on in vitro experiments and the identification of key PK-TD parameters, allows for a more efficient prediction of drug toxicity while maintaining high predictive accuracy. Therefore, the in vitro parameter-based approach for assessing in vivo cardiotoxicity serves as a valuable early screening tool for identifying the cardiotoxic potential of compounds.

However, there are some limitations in our study. Firstly, the data utilized for the development of the translational method were obtained from the literature, introducing potential interstudy variability that may impact prediction accuracy. Secondly, our analysis primarily focused on the direct effects of drugs on cardiomyocytes, including viability, mitochondrial function, and contractility, as well as LVEF decline as a key clinical endpoint. However, broader cardiovascular toxicities, such as hypertension and QT prolongation, particularly relevant for VEGF inhibitors, were not fully addressed. Variations in blood pressure and heart rate can significantly influence cardiac function, potentially leading to a decline in left ventricular ejection fraction [57,58]. Thirdly, the current model, constrained to a single time point for assessing drug effects on cardiomyocytes, fails to account for the temporal aspect, which is crucial in determining drug toxicity. Furthermore, the model may not fully capture patient-to-patient variability. Future iterations of the model should incorporate temporal dynamics and refine predictive methodologies to enhance translational accuracy and improve cardiotoxicity risk assessment.

## 4. Materials and Methods

### 4.1. Data Collection

#### 4.1.1. PK Models of TKIs

A literature search was performed to collect PK models for the 21 TKIs using the keywords “human”, “population pharmacokinetic”, and/or “pharmacokinetic” from the PubMed database and reviews from U. S. Food and Drug Administration (FDA). The collected parameters of the PK models are presented in Table S2. The plasma pharmacokinetics of the investigated TKIs were simulated based on these models.

#### 4.1.2. In Vitro Data on TKI Cardiotoxicity with hiPSC-CMs

Data on cardiomyocyte viability and contractility were collected from a high-throughput study using human induced pluripotent stem cell-derived cardiomyocytes [12]. This study evaluated the cardiotoxicity of 21 FDA-approved tyrosine kinase inhibitors (TKIs), including afatinib, axitinib, bosutinib, cabozantinib, crizotinib, dabrafenib, dasatinib, erlotinib, gefitinib, ibrutinib, imatinib, lapatinib, nilotinib, pazopanib, ponatinib, regorafenib, sorafenib, sunitinib, trametinib, vandetanib, and vemurafenib. And drug-induced changes in cell survival, ATP levels, and contraction force were assessed. These results were extracted using Engauge Digitizer 11.1.

#### 4.1.3. Clinical Incidence of TKI-Induced Cardiac Dysfunction

TKI-induced cardiotoxicity can manifest as cardiac dysfunction (heart failure), QT interval prolongation, or myocardial infarction [12,57,59]. This study focuses on cardiac dysfunction and heart failure that were determined by the reduced left ventricular ejection fraction (LVEF). Clinical incidence of cardiac dysfunction related to these TKIs was obtained from FDA reviews and PubMed searches using the keywords “LVEF”, “heart failure”, and “cardiac dysfunction”. Specifically, only clinical studies linking cardiac dysfunction to reduced LVEF were included. Detailed incidence rates of TKI-induced cardiac dysfunction are presented in Table S3.

### 4.2. In Vitro TD Model

**Figure 9:**
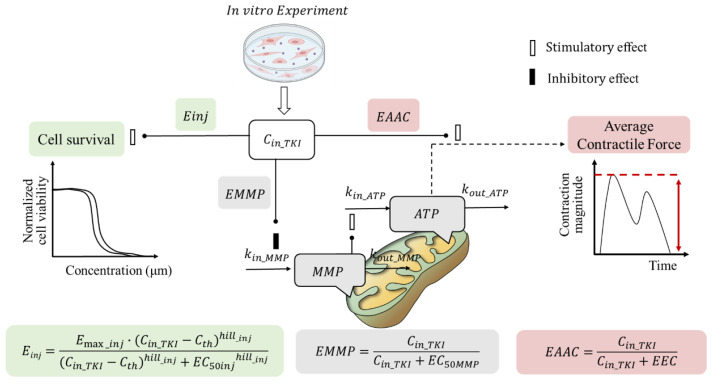
Schematic diagram of TKI-induced cardiomyocyte injury. C_in_TKI_ is the effective concentration of intracellular TKIs, and Cth is the threshold concentration of TKIs. E_max_inj_ is the maximum effect coefficient; hill__inj_ is the hill index. EC_50inj_ is TKI concentration for half the maximum cell injury. MMP, is the mitochondrial membrane potential; kin__ATP_ and kin__MMP_ are the zero-order production rate constants for ATP and MMP, respectively. kout__ATP_ and kout__MMP_ are the first-order elimination rate constants for ATP and MMP, respectively. EC_50MMP_ is the TKI concentration at which MMP production reaches half the maximum; EEC, TKI concentration at which average contractile force reaches half the maximum. E_inj_, EMMP, and EAAC are the effect of drug exposure on cell survival, MMP, and average contractile force.

The in vitro toxicodynamic (TD) model used in this study was adapted from previous work [19]. TKIs-induced cardiac dysfunction may result from effects on cardiomyocyte viability, mitochondrial damage, and/or contractility [6,7]. This TD model includes three sub-models to evaluate drug effects on cardiomyocyte viability, ATP levels, and contractility (Figure 9), reflecting three mechanisms of cardiac dysfunction: (1) apoptosis or reduced cell viability at drug concentrations above a certain threshold, (2) impaired energy production affecting cardiac contraction, and (3) disrupted cardiomyocyte function or structure increasing energy demand.

#### 4.2.1. Sub-Model for Cardiomyocyte Viability

Tyrosine kinase inhibitors may impair cardiomyocyte viability by inhibiting critical signaling pathways for cell survival and maintenance [7,8]. It is assumed that TKI-induced cardiomyocyte dysfunction may sustain contractility in early apoptosis stages. An indirect effect model describes the impact of TKIs (Equation (1)). The survival number of cardiomyocytes includes normal and injured populations (Equation (8)). A threshold concentration (Cth) is introduced to describe the nontoxic range, below which the drug has no significant toxic effect. The governing differential equations for the drug’s impact on cardiomyocyte viability are as follows (Equations (1)–(7)):

(1) $$E_{inj}=\frac{E_{max\_inj}\cdot {\left(C_{in\_TKI}-C_{th}\;\right)}^{{hill}_{\_inj}}}{{\left(C_{in\_TKI}-C_{th}\;\right)}^{{hill}_{\_inj}}+{{EC}_{50inj}}^{{hill}_{\_inj}}}$$

(2) $$\frac{dF_{nor}}{dt}=-E_{inj}\cdot F_{nor}\cdot k_{inj}$$

(3) $$\frac{dF_{inj\_1}}{dt}=E_{inj}\cdot F_{nor}\cdot k_{inj}-\frac{F_{inj\_1}}{\tau }$$

(4) $$\frac{dF_{inj\_2}}{dt}=\frac{F_{inj\_1}}{\tau }-\frac{F_{inj\_2}}{\tau }$$

(5) $$\frac{dF_{inj\_3}}{dt}=\frac{F_{inj\_2}}{\tau }-\frac{F_{inj\_3}}{\tau }$$

(6) $$F_{inj}=F_{inj\_1}+F_{inj\_2}+F_{inj\_3}\;$$

(7) $$\frac{dF_{dead}}{dt}=\frac{F_{inj\_3}}{\tau }$$

(8) $$Survival\;number=F_{nor}+F_{inj}\;$$

where E_inj_ is the effect of drug exposure on cell survival. k_inj_ is the the rate constant for the conversion of normal cells to injured cells. E_max_inj_ is the maximum effect coefficient, fixed at 1; hill__inj_ is the hill index. C_in_TKI_ is the effective concentration of the drug in the medium. Cth is the threshold concentration to describe the nontoxic effect of the investigated drug under the defined threshold. F_nor_, F_inj_, and F_dead_ are the percentages of normal, injured, and dead cells to the total number of cardiomyocytes, respectively. τ is the average transit time of programmed death of cardiomyocytes. F_inj_1_, F_inj_2_, and F_inj_3_ are the fraction of early-, middle-, and late-phase injured cardiomyocyte populations, respectively.

#### 4.2.2. Sub-Model for Cardiomyocyte ATP and MMP

Mitochondrial damage and cardiac fibrosis may underlie TKI-induced cardiotoxicity [8]. The reduction in average contraction force (AAC) due to mitochondrial damage can be mechanistically linked to decreased ATP levels and mitochondrial membrane potential (MMP), as shown in Equations (9)–(11).

(9) $$EMMP=\frac{C_{in\_TKI}}{C_{in\_TKI}+{EC}_{50MMP}}\;$$

(10) $$\frac{dMMP}{dt}=k_{in\_MMP}\cdot \left(1-EMMP\right)-k_{out\_MMP}\cdot MMP\;$$

(11) $$\frac{dATP}{dt}=k_{in\_ATP}\cdot {MMP}^{n}-k_{out\_ATP}\cdot ATP\;$$

where EMMP is the effect of drug exposure on MMP. kin__ATP_ and kin__MMP_ are the zero-order production rate constants for ATP and MMP, respectively. kout__ATP_ and kout__MMP_ are the first-order elimination rate constants for ATP and MMP, respectively. EC_50MMP_ is the concentration of TKIs at which MMP production reaches half the maximum. To describe the stimulation of MMP on ATP, n is estimated according to the effect of doxorubicin on hiPSC-CMs in our previous study [19].

#### 4.2.3. Sub-Model for Contractility of Cardiomyocytes

In the case of AAC reduction due to cardiac fibrosis, it is assumed that a higher ATP demand is needed to achieve the same AAC as before drug treatment. Consequently, the ATP at half the maximum mean contractility of cardiomyocytes (ATP_50_) is considered proportional to drug exposure (Equations (12)–(14)).

(12) $$EAAC=\frac{C_{in\_TKI}}{C_{in\_TKI}+EEC}\;$$

(13) $${ATP}_{50}=\frac{{ATP}_{50\_ref}}{(1-EAAC)\;}\;$$

(14) $$AAC=\frac{ATP\cdot (1+{ATP}_{50\_ref})}{ATP+{ATP}_{50}\;}\;$$

AAC is the average contractile force. EAAC is the effect of drug exposure on AAC. ATP_50_ref_ is the ATP level that exerts half of maximal contraction (ATP_50_) before TKI treatment, which is from a previous study [19]. EEC is the TKI concentration at which AAC reaches half the maximum. In model parameter optimization, EAAC is fixed as 0 once it cannot be correctly estimated.

#### 4.2.4. Estimation of TD Model Parameters

The collected observations on survival fraction, ATP levels, and contractile force, which were used to estimate model parameters related to cell viability, mitochondrial function, and contractility in the TD model. Some parameters, such as τ, kin__MMP_, kin__ATP_, and n, were adapted from the previous study [19], while this study focused on parameters related to the indirect effects of TKIs on cell injury and contractility, including k_inj_, EC_50inj_, hill_inj_, EC_50MMP_, and EEC.

### 4.3. Quantitative Systems Pharmacology (QSP) Model

The QSP model was modified to depict the systemic response of TKIs to cardiomyocyte injury in vivo, based on a previous study [23]. Systemic parameters, including kout_LVEDV, kout_TPR, kout_SV, kout_HR, LVEDV, and index_SV, were derived from earlier findings [23]. Initial values for SV, TPR, LVEDV, HR, MAP, and LVEF vary across cardiovascular disease states. The parameter FB_LVESV represents the feedback regulation coefficient of LVESV on LVEDV, with specific values for healthy, hypertensive, and hypertrophic cardiovascular conditions, as listed in Table 2.

Myocardial contractility reflects the total capacity of surviving cardiomyocytes (Equation (15)). In the TD model, TKIs impair cardiomyocyte viability (Equations (1)–(8)) and ATP levels, reducing myocardial contractile function (Equation (14)). In the QSP model, the effect of TKIs on cardiomyocytes (E_TKI_) replaces the drug’s effect on stroke volume (E_EP_SV_) [23] in the TD model (Equation (15)). This leads to decreased stroke volume (SV) and a subsequent reduction in LVEF in the QSP model.

(15) $${\;E}_{TKI}=Survival\;number\cdot AAC\;$$

### 4.4. IVIVE of TKI-Induced Cardiotoxicity

#### 4.4.1. Quantitative Prediction of the Incidence of Cardiac Dysfunction

Virtual clinical studies using the QSP-PK-TD model quantitatively predicted the incidence rates of TKI-induced cardiotoxicity. Pharmacokinetic models and parameters for each TKI were integrated into the QSP-PK-TD framework. The cellular toxicodynamic model described cardiotoxic effects based on in vitro tests. The QSP model simulates changes in the left ventricular ejection fraction across different populations.

Building upon the previous IVIVE method (Supplementary Methods, Figure S4), this study simulated the incidence of cardiac dysfunction in healthy individuals, as well as those with hypertension and dilated cardiomyopathy [19,23]. A total of 5000 virtual patients were generated for each population using Monte Carlo simulations to estimate TKI-induced cardiotoxicity incidence rates. The prevalence of hypertension is 35%, and dilated cardiomyopathy is 0.03% [65,66]. The patient ratios were 64.97% cardiovascular healthy, 35% hypertensive, and 0.03% with dilated cardiomyopathy. In hypertensive patients, the mean arterial pressure (MAP) was set at 106 mmHg or above [60], and in those with dilated cardiomyopathy, the left ventricular end-diastolic volume (LVEDV) was set at 146 mL or above [53].

Changes in the left ventricular ejection fraction induced by tyrosine kinase inhibitors after one year were simulated to assess cardiotoxicity, the simulation times were chosen based on reported durations of cardiotoxic drug in the literature [19,23]. The International Cardio-Oncology Society [67] defines drug-induced cardiotoxicity as a reduction in LVEF of more than 10% from baseline to below 50%, or a decrease of 20% [68]. The total incidence of TKI-induced cardiotoxicity was calculated by summing the product of each population’s ratio and their incidence rates, as shown in Equation (16).

(16) $$Incidence=\sum_{i=1}^{n}\left(Inicidence\;of\;{Population}_{i}\cdot {Proportion\;of\;Population}_{i}\right)$$

#### 4.4.2. Qualitative Identification of Cardiotoxic Drugs

The QSP model identified E_inj_, MMP, and AAC as key drivers of cardiac dysfunction and toxic targets of TKIs. Since estimated threshold concentrations of E_inj_ exceeded the C_max_ for most TKIs, it was excluded as a factor to describe drug-induced cardiotoxicity. Instead, drug effects of TKIs on MMP and AAC were assessed as potential indicators of cardiotoxicity. Since the cumulative effects on MMP and AAC stabilized over a dosing cycle upon reaching a steady state, the area under the effect curve for EMMP (AEMMP) and EAAC (AEAAC) was calculated. This analysis was used to distinguish cardiotoxic from noncardiotoxic TKIs. Recommended dosing regimens for the TKIs are provided in Table S4.

### 4.5. Factors Affecting Effective Concentration in IVIVE

Quantifying the difference in effective drug exposure between in vitro and in vivo conditions is critical for successful IVIVE. In in vitro studies, the added drug directly interacts with cultured cardiomyocytes, and the concentration in the medium is often regarded as the effective drug concentration. However, in vivo studies commonly use plasma drug concentration as a surrogate for the actual target tissue concentration when describing the exposure–response relationship. To improve IVIVE accuracy for TKI-induced cardiotoxicity, parameters related to drug disposition, such as protein binding and tissue distribution, were introduced as adjustment factors. These factors help refine the correlation between in vitro and in vivo effective drug concentrations, allowing for more precise prediction of cardiotoxicity.

#### 4.5.1. Plasma Protein Binding

The unbound plasma drug concentration indicates the active drug level in cardiomyocytes. In the in vitro–in vivo extrapolation, the effective drug concentration in the myocardium (C_eff,vivo_) is derived from the product of the simulated plasma drug concentration and the free fraction in plasma (Table S5).

(17) $$C_{eff,vivo}=C_{plasma,free}=C_{total,plasma}\cdot f_{u,plasma}\;$$

where C_total,plasma_ are the total drug concentrations in plasma and f_u,plasma_ are the plasma drug-free fractions.

#### 4.5.2. Tissue–Plasma Partition

Drug accumulation in cardiomyocytic tissues is another key factor contributing to the difference between plasma and target exposure. The active drug concentration in the heart is assumed to be the product of plasma concentration and the tissue–plasma partition coefficient (K_p_). Koichiro et al. [69] found that K_p_ values simulated using the Poulin and Theil method based on in silico data are more suitable for estimating toxicokinetic or internal exposure. For drugs without reported tissue exposure data, this method was employed to estimate K_p_ (Equation (20)) [46]. K_p_ is calculated as the ratio of drug exposure in tissue to that in plasma, based on available data. The physicochemical properties of the TKIs are summarized in Table S6 and predicted K_p_ values are listed in Table S7.

(18) $$C_{eff,vivo}=C_{plasma}\cdot K_{p}\;$$

(19) $$K_{p\_observation}=\frac{{AUC}_{heart}}{{AUC}_{plasma}}\;$$

(20) $$K_{p\_prediction}=\frac{{[K}_{vo:w}\cdot (V_{net}+0.3\cdot V_{pht})]+{[V}_{wt}+0.7\cdot V_{pht}]}{{[K}_{vo:w}\cdot (V_{nep}+0.3\cdot V_{php})]+{[V}_{wp}+0.7\cdot V_{php}]}\;$$

where K_vo:w_ is the oil–water partition coefficient of the drug. V_net_ is the tissue volume fraction of neutral phospholipids. V_nep_ is the plasma volume fraction of neutral phospholipids. V_pht_ is the tissue volume fraction of phospholipid. V_php_ is the plasma volume fraction of phospholipid. V_wt_ is the tissue volume fraction of water. V_wp_ is the plasma volume fraction of water.

#### 4.5.3. Drug Heterogeneous Distribution

In monolayer 2D cell cultures, cardiomyocytes are uniformly exposed to drugs, while in 3D cardiomyocytic tissues, drug access is heterogeneous. This difference contributes to a translational barrier between in vitro and in vivo conditions [49]. A reduced toxic profile has been noted in 3D hiPSC-CMs-based cardiotoxicity tests compared to 2D cultures [12,17,50]. To correct for this discrepancy, the heterogeneous distribution (H) of the drug in vivo is adjusted using a heterogeneity parameter. This parameter is calculated as the ratio of the IC_50_ in 2D culture (IC_50,2D_) to that in 3D culture (IC_50,3D_), as shown in Equation (21). For drugs lacking a reported IC_50,3D_, it is assumed that IC_50,3D_ equals IC_50,2D_.

(21) $$C_{eff,vivo}=C_{plasma}\cdot \frac{{IC}_{50,2D}}{{IC}_{50,3D}}\;$$

IC_50,2D_ and IC_50,3D_ are the concentrations that reach the half of maximal drug effect in hiPSC-CMs, based on an in vitro study (Table S8).

### 4.6. Sensitivity Analysis

Sensitivity analyses were performed using a single-method approach to evaluate the impact of both systemic and drug-specific parameters on left ventricular ejection fraction. Systemic parameters include index_SV, FB_LVESV, LVEDV, kout_SV, kout_LVEDV, kout_TPR, koutHR, n, k_in_ATP_, k_in_MMP_, and τ. Drug specific parameters include fu, K_p_, H, Ka, CL, Vc, Vp, Q, k_inj_, hill__inj_, EC_50inj_, EC_50MMP_, and EEC. The parameter range was determined based on reported physiological variability and previous studies [19]. Specifically, LVEDV and index_SV were set within the ranges of 45–145.8 [53,70] and 0.5–0.7 [62], respectively. FB_LVESV was adjusted to 0.66–1.07 times the typical value from the previous model (0.003473) [19], while all other parameters varied between one-third and three times their typical values.

The sensitivity of the parameter was set as the changes in typical values of the parameters (∆pi) and changes in the LVEF (∆LVEFchange) caused by changes in typical values during one year of TKI administration. Sensitivity was calculated by Equation (22).

(22) $$Sensitivity=\frac{p_{i}}{LVEFchange}\cdot \frac{∆LVEFchange}{∆p_{i}}\;$$

(23) $$LVEFchange=\frac{LVEF-{LVEF}_{initial}}{{LVEF}_{initial}}\cdot 100$$

(24) $$∆LVEFchange={LVEF}_{one\;year}-{LVEF}_{initial}$$

LVEF_initial_ represents the LVEF value at the initial time. LVEF_one year_ represents the LVEF value at one year.

### 4.7. Data Analysis

Literature data were extracted with Enguage Digitizer 10.8. Model fitting and optimization were performed using Monolix Suite 2021 R2 (Lixoft, Antony, France). Simulations were conducted in R 4.2.0 with the mrgsolve package, and plots were generated with ggplot2. Sensitivity analysis was conducted using the dplyr package.

## 5. Conclusions

This study successfully constructed mechanism-based PD models for 21 TKIs through high-throughput cardiotoxicity screening, integrating multiple cardiotoxic mechanisms. By combining the PK and QSP models, the established QSP-PK-TD model was validated for in vitro to in vivo extrapolation. During this process, the model focused on evaluating discrepancies in effective drug concentrations caused by differences in in vitro and in vivo physiology. Although the overestimation of cardiotoxicity for certain drugs due to overpredicted tissue–plasma partitioning was not specifically addressed, the model’s qualitative differentiation and quantitative translational accuracy were further refined by incorporating key factors such as plasma protein binding, tissue–plasma partitioning, and drug distribution heterogeneity in high-throughput testing using hiPSC-CMs. Additionally, we identified two key PK-TD parameters—AEMMP and AEAAC—as crucial for identifying cardiotoxic TKIs through in vitro experiments. Overall, this study provides a method for assessing in vivo cardiotoxicity using parameters derived from in vitro experiments. By integrating the QSP-PK-TD model and accounting for factors that influence drug exposure, such as plasma protein binding, tissue–plasma partitioning, and drug distribution heterogeneity, we can extract critical information from in vitro data, facilitating the translation from in vitro to in vivo. This approach offers new insights for evaluating the cardiotoxicity of tyrosine kinase inhibitors and provides a valuable tool for drug clinical development and safety assessment.

## Figures and Tables

**Table 1:** Parameters for the TD model of tyrosine kinase inhibitors.

Drug	$k_{inj}$ (μM) (×10^−2^)	$EC_{50_{inj}}$ (μM)	${hill}_{\_inj}$	$C_{th}$ (μM)	$EC_{50_{MMP}}$ (μM)	EEC (μM)
Afatinib	15	17.62	3	4.16	4.62	0.41
Axitinib	0.32	4	1.04	4.7	50.15	/
Bosutinib	12	14.57	3	5.16	9.53	/
Cabozantinib	2.7	64.72	0.67	3.7	10.84	/
Crizotinib	4.4	12.05	1.61	1.7	10.8	44.24
Dabrafenib	1.9	55.25	1.18	28.6	45.75	/
Dasatinib	3.1	19.99	0.76	9.6	13.26	2.76
Erlotinib	1.9	93.47	0.76	5.96	5.54	/
Gefitinib	7.4	44.59	1.91	5.16	10.64	10.47
Ibrutinib	27	26.39	3	4.16	5.63	2.50
Imatinib	18	72.74	3	19.8	16.41	32.97
Lapatinib	1.3	27.39	1.38	24.2	69.6	1.80
Nilotinib	6.8	97.37	1.05	2.2	7.16	28.16
Pazopanib	1.5	72.36	0.69	13	21.49	/
Ponatinib	11	11.6	1.54	0.46	2.54	2.57
Regorafenib	4.4	5.38	1.58	2.7	3.2	16.27
Sorafenib	4.5	4.36	3	1	3.31	0.79
Sunitinib	10	22.02	3	2.2	5.01	1.42
Trametinib	1.3	24.77	2.61	15.4	12.59	0.94
Vandetanib	7.1	79.89	1.05	1.38	10.72	0.94
Vemurafenib	2.6	44.65	0.87	2.2	15.46	31.32

**Table 2:** Parameters of the quantitative systemic pharmacology model in three populations.

	Healthy	Hypertension	Dilated Cardiomyopathy
MAP_0_	94.92	106 [60]	91.54
LVEDV_0_	113 [61]	100.95	146 [53]
Index_SV_0_	0.6 [62]	0.6	0.55 [63]
FB__LVESV_	0.0042	0.0045	0.0028
CV_MAP	0.1	0.2	0.3
CV_LVEDV	0.1	0.2	0.2
CV_Index_SV_0_	0.05	0.05	0.05
CV_FB__LVESV_	0.124	0.124	0.124
SV_0_	67.8	60.57	80.3
TPR_0_	0.02	0.025	0.02
HR_0_	70	70	57 [64]

## Data Availability

The original contributions presented in this study are included in the article/supplementary material. Further inquiries can be directed to the corresponding authors.

## Supplementary Materials

The following supporting information can be downloaded at: https://www.mdpi.com/article/10.3390/ph18040450/s1. Supplementary Methods: In vitro to in vivo extrapolation (IVIVE). Figure S1. Incidence of cardiac dysfunction based QSP-PK-TD model in healthy, hypertension and dilated cardiomyopathy populations. Figure S2. The Pearson correlation analysis between EEC and EC50MMP of toxic-TKIs and sensitivity indices. Figure S3. Image of EAAC and EMMP over time. Figure S4. A summary of the IVIVE method based on the QSP-PK-TD model. Table S1. Intracellular effective (C_eff_TKIs_) and threshold (C_th_) concentration of cardiotoxic TKIs based on QSP-PK-TD model. Table S2. The population PK parameters of TKIs. Table S3. Summary literature for clinical cardiac dysfunction incidence of tyrosine kinase inhibitors. Table S4. Summary literature for dosage regimen of tyrosine kinase inhibitors. Table S5. Drug free fraction of tyrosine kinase inhibitors in plasma. Table S6. The physicochemical properties of tyrosine kinase inhibitors. Table S7. Tissue partition coefficients of tyrosine kinase inhibitors. Table S8. Summary literature for IC_50,3D_ and IC_50,2D_ of tyrosine kinase inhibitors.
