# Supplementary material for: Bridging the Gap Between hiPSC-CMs Cardiotoxicity Assessment and Clinical LVEF Decline Risk: A Case Study of 21 Tyrosine Kinase Inhibitors
Source: Pharmaceuticals (Basel). 2025 Mar 23;18(4):450. doi: 10.3390/ph18040450 (PMC12030206; doi:10.3390/ph18040450)
Supplement: Supplementary file 1 [file pharmaceuticals-18-00450-s001.zip › pharmaceuticals-3505712-supplementary.pdf]

Supplementary

Supplementary Methods: *In vitro* to *in vivo* extrapolation (IVIVE)

Figures

Figure S1. Incidence of cardiac dysfunction based QSP-PK-TD model in healthy, hypertension and dilated cardiomyopathy populations.

Figure S2. The Pearson correlation analysis between EEC and $EC_{50MM}P$ of toxic-TKIs and sensitivity indices.

Figure S3. Image of EAAC and EMMP over time.

Figure S4. A summary of the IVIVE method based on the QSP-PK-TD model.

Tables

Table S1. Intracellular effective (C_{eff_TKIs}) and threshold (C_{th}) concentration of cardiotoxic TKIs based on QSP-PK-TD model.

Table S2. The population PK parameters of TKIs.

Table S3. Summary literature for clinical cardiac dysfunction incidence of tyrosine kinase inhibitors.

Table S4. Summary literature for dosage regimen of tyrosine kinase inhibitors.

Table S5. Drug free fraction of tyrosine kinase inhibitors in plasma.

Table S6. The physicochemical properties of tyrosine kinase inhibitors

Table S7. Tissue partition coefficients of tyrosine kinase inhibitors.

Table S8. Summary literature for $IC_{50,3D}$ and $IC_{50,2D}$ of tyrosine kinase inhibitors.

Method: *In vitro* to *in vivo* extrapolation (IVIVE)

The *in vitro* to *in vivo* extrapolation method was employed to translate drug effects observed in *in vitro* experiments into predictions of *in vivo* outcomes [1]. In the previous study, the quantitative systems pharmacology (QSP) model [2] was developed to assess drug effects across different cardiovascular populations by incorporating key physiological components, including preload, determined by LVEDV, MAP, and myocardial compliance; afterload, represented by total peripheral resistance (TPR); and myocardial contractility, influenced by cardiac energy metabolism and myocardial compliance. This model classified patients into three cardiovascular subgroups. Healthy individuals exhibited relatively stable LVEF before and after treatment, indicating greater tolerance to doxorubicin. Hypertensive patients experienced increased vascular resistance, which elevated afterload and led to a more pronounced decline in LVEF. Patients with dilated cardiomyopathy (enlarged LVEDV) suffered from excessive ventricular dilation, impairing cardiac contractility and resulting in the most severe LVEF reduction.

In this study, the IVIVE methodology was adapted to integrate population pharmacokinetics (PK) models, toxicodynamic (TD) model, and QSP model to extrapolate *in vitro* experimental data to *in vivo* predictions. The PK model predicts drug exposure in plasma, while the TD model evaluates direct drug effects on cardiomyocytes, including mitochondrial function, cell viability, and contractility. Additionally, the QSP model simulates systemic responses to drug-induced cardiac injury, particularly changes in left ventricular ejection fraction across different patient populations. A summary of the overall IVIVE methodology used in this study is provided in Figure S4.

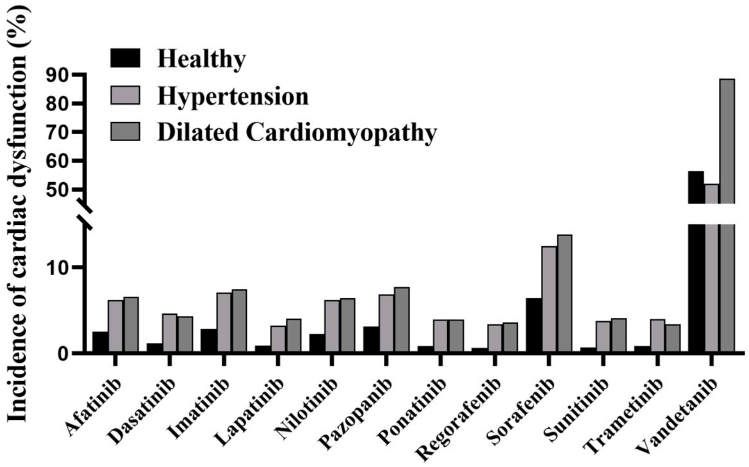

Figure S1. Incidence of cardiac dysfunction based QSP-PK-TD model in healthy, hypertension and dilated cardiomyopathy populations.

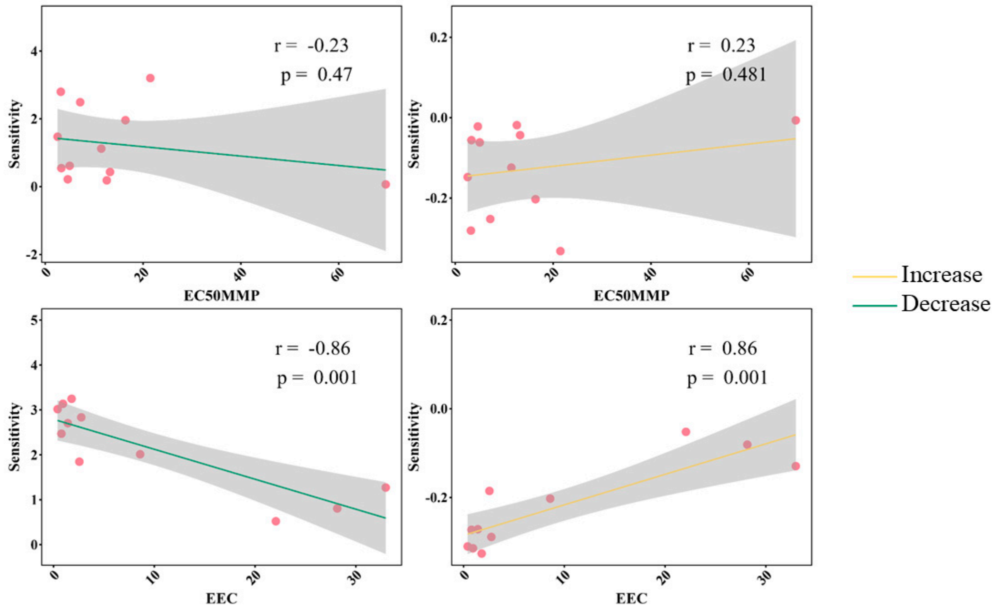

Figure S2. The Pearson correlation analysis between EEC and EC_{50MMP} of toxic-TKIs and sensitivity indices.

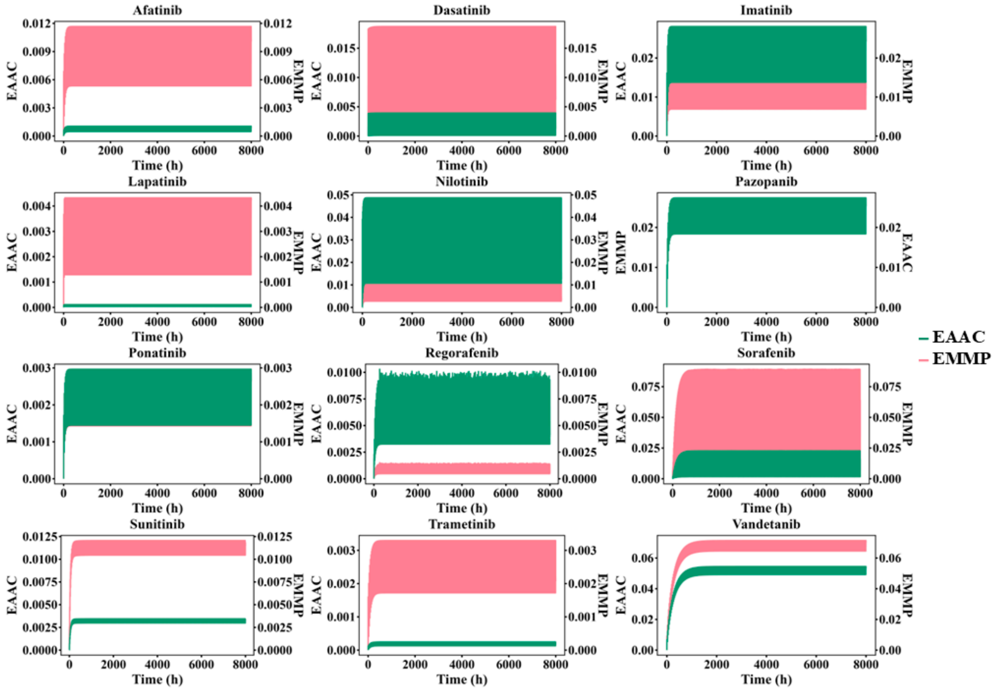

Figure S3. Image of EAAC and EMMP over time.

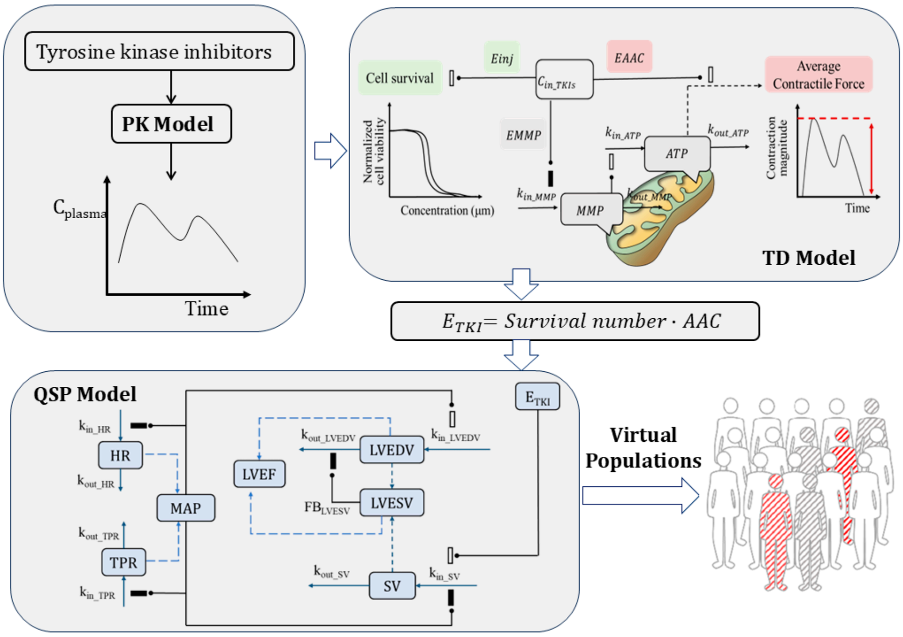

Figure S4. A summary of the IVIVE method based on the QSP-PK-TD model.

Table S1. Intracellular effective (C_{eff_TKI}) and threshold (C_{th}) concentration of cardiotoxic TKIs based on QSP-PK-TD model.

	C_{eff_TKI} (μmol/L)	C_{th} (μmol/L)
Afatinib	0.01	4.16
Dasatinib	0.05	9.6
Imatinib	0.47	19.8
Lapatinib	0.008	24.2
Nilotinib	0.15	2.2
Pazopanib	0.61	13
Ponatinib	0.0076	0.46
Regorafenib	0.01	2.7
Sorafenib	0.077	1
Sunitinib	0.047	2.2
Trametinib	0.0003	15.4
Vandetanib	1.32	1.38

C_{eff_TKIs} represents the heterogenous distribution of tissue free drug as effective drug exposure.

Table S2. The population PK parameters of TKIs.

	Ka(1/h)	CVka	ktr	Tlag(h)	CVTlag	CL(L/h)	CVCL	Vc(L)	CVVc	Q(L/h)	CVQ	Vp(L)	CVVp	F	CVF	Paper
Afatinib	0.25	0.77	/	/	/	42.3	/	456	/	77.52	/	1132	/	1	0.48	[3]
Axitinib	0.53	0.71	/	0.46	/	17	0.52	45.3	0.31	1.74	0.64	45.9	1.03	0.465	/	[4]
Bosutinib	0.61	1.04	/	0.87	/	120	0.60	4917	0.48	56.38	/	388300	2.11	1	/	[5]
Cabozantinib	0.57	0.66	/	0.46	/	2.23	0.46	81.5	1.03	14.20	/	213	/	0.675	/	[6]
Crizotinib	0.82	1.28	/	0.65	/	102	0.31	2390	0.48	44.40	/	2200	/	1	/	[7]
Dabrafenib	1.88	1.60	/	0.48	/	17	0.59	70.3	0.53	3.30	0.99	154	/	0.555	/	[8]
Dasatinib	0.26	0.17	/	/	/	126	0.24	166	0.75	11.10	/	1296	/	1	/	[9]
Erlotinib	1.48	/	/	/	/	3.8	0.42	166	0.54	/	/	/	/	1	/	[10]
Gefitinib	0.39	0.74	/	/	/	21	0.79	934	0.83	46.9	0.78	1500	1.30	1	/	[11]
Ibrutinib	/	/	/	0.32	0.22	56.8	0.59	309	0.64	73.40	1.49	270	0.75	0.039	/	[12]
Imatinib	1.03	0.82	/	/	/	7.29	0.19	202	0.6	/	/	/	/	1	/	[13]
Lapatinib	0.12	0.12	/	/	/	19.36	0.37	124	0.52	/	/	/	/	1	/	[14]
Nilotinib	/	/	/	0.75	/	12.8	0.27	56	1.29	103	/	247	0.72	1	0.62	[15]
Pazopanib	0.98	0.21	/	/	/	0.458	0.71	22.3	0.25	/	/	/	/	1	/	[16]
Ponatinib	4.41	/	1.30	3.93	/	34.28	0.48	838.6	0.42	17.21	/	347.4	/	0.534	/	[17]
Regorafenib	0.48	0.36	0.48	0.36	/	4.02	0.35	10.7	/	11.00	/	162	/	1	/	[18]
Sorafenib	2.53	0.62	/	/	/	8.13	0.18	213	0.69	/	/	/	/	1	/	[19]
Sunitinib	0.38	0.88	/	0.64	/	24.1	0.34	1070	0.24	0.28	/	63.8	/	1	/	[20]
Trametinib	0.91	/	/	0.71	/	5.83	0.30	61.9	/	64.90	0.80	417	/	1	/	[21]
Vandetanib	0.83	0.11	/	/	/	7.26	0.08	1280	0.08	10.70	/	616	/	1	/	[22]
Vemurafenib	0.19	1.01	/	/	/	1.3	0.32	106	0.66	/	/	/	/	0.85	/	[23]

ka, drug absorption rate constant. ktr, the drug transduction rate constant. Tlag, the delay time of drug absorption. CL, drug clearance rate. Vc, central compartment volume. Vp, peripheral compartment volume. Q, blood flow rate. F, bioavailability.

Table S3. Summary literature for clinical cardiac dysfunction incidence of tyrosine kinase inhibitors

Drug	Incidence of cardiac dysfunction (%)	Patience number	Reference
Afatinib	0-5.3	-	[24]
	5.3	38	[25]
Dasatinib	4	911	[26]
	2-4	-	[27]
Imatinib	1.7	1276	[28]
	0.04	2327	[29]
	0.2-2.7	-	[27]
Lapatinib	1.5	2311	[30]
	1.76	2057	[31]
	0.2-1.5	-	[27]
Nilotinib	1	24444	[27]
	13.4	277	[32]
Pazopanib	13	557	[33]
	7-11	-	[27]
Ponatinib	7	449	[32]
Regorafenib	0.3-5.6	2056	[34]
Sorafenib	4-8	-	[27]
	3.3-6.6	510	[35]
	13.1-18.6	510	[35]
Sunitinib	2.7	224	[36]
	2.7-19	-	[27]
	11	75	[33]
	5.2	513	[35]
	3.7-7.2	-	[35]
Trametinib	11	139	[37]
	3	97	[38]
	7	211	[39]
	11	329	[39]
Vandetanib	0.9	-	[40]

Table S4. Summary literature for dosage regimen of tyrosine kinase inhibitors.

Number	Drug	Dosage regimen	Administration times	Reference
1	Afatinib	40 mg, QD	27 times	[41]
2			28 times	[42]
3			21 times	[43]
4			21 times	[44]
5			single	[45]
6			21 times	[46]
7			21 times	[47]
8	Axitinib	5 mg, BID	single	[48]
9			single	[49]
10			single	[50]
11			single	[51]
12			single	[52]
13	Bosutinib	500 mg, QD	single	[53]
14			single	[54]
15			single	[55]
16			single	[56]
17	Cabozantinib	140mg	single	[57]
18			single	[58]
19			single	[59]
20			single	[60]
21	Crizotinib	250 mg, BID	single	[61]
22			single	[62]
23			single	[63]
24			54 times	[64]
25			single	[65]
26	Dasatinib	100 mg, QD	single	[66]
27			28 times	[67]
28			single	[68]
29			single	[69]
30			single	[70]
31			single	[71]

32	Dabrafenib	150 mg, BID	single	[72]
33			single	[73]
34			single	[74]
35	Erlotinib	150 mg, QD	single	[75]
36			14 times	[76]
37			single	[77]
38			single	[78]
39			8 times	[79]
40			single	[80]
41			14 times	[81]
42			22 times	[82]
43			single	[83]
44			single	[84]
45	Gefitinib	250 mg, QD	single	[85]
46			single	[86]
47			21 times	[87]
48			single	[88]
49			single	[89]
50			single	[90]
51			single	[91]
52	Ibrutinib	560 mg, QD	single	[92]
53			20 times	[93]
54			single	[94]
55			single	[95]
56	Imatinib	400 mg, QD	28 times	[96]
57			single	[97]
58			8 times	[98]
59			30 times	[99]
60			single	[100]
61			7 times	[101]
62	Lapatinib	1250 mg, QD	14 times	[102]
63			7 times	[103]
64			7 times	[104]
65			15 times	[105]

66	Nilotinib	400 mg, BID	single	[106]
67			29 times	[107]
68			single	[108]
69			single	[109]
70	Pazopanib	800 mg, QD	21 times	[110]
71			14 times	[111]
72			15 times	[112]
73			single	[113]
74	Ponatinib	45 mg, QD	single	[114]
75			single	[115]
76			single	[116]
77			single	[117]
78	Regorafenib	160 mg, QD	20 times	[118]
79			42 times	[119]
80			14 times	[120]
81			13 times	[121]
82	Sorafenib	400 mg, BID	13 times	[122]
83			41 times	[123]
84			15 times	[124]
85	Sunitinib	50 mg, QD	14 times	[125]
86			single	[126]
87			42 times	[127]
88			28 times	[128]
89			single	[129]
90			28 times	[130]
91	Trametinib	2 mg, QD	single	[131]
92			single	[132]
93			single	[133]
94			single	[134]
95			single	[135]
96	Vandetanib	300 mg, QD	43 times	[136]
97			single	[137]
98			single	[138]
99			29 times	[139]

100			39 times	[140]
101	Vemurafenib	960 mg, QD	single	[141]
102			43 times	[142]
103			29 times	[72]
104			single	[73]

QD, once a day; BID, twice a day.

Table S5. Drug free fraction of tyrosine kinase inhibitors in plasma.

Drug	$f_{u, plasma}$ (%)	Reference
Afatinib	5	DrugBanK
Axitinib	1	DrugBanK
Bosutinib	6	DrugBanK
Cabozantinib	0.3	DrugBanK
Crizotinib	9	DrugBanK
Dabrafenib	0.3	DrugBanK
Vemurafenib	0.001	[143]
Erlotinib	7	DrugBanK
Gefitinib	0.1	DrugBanK
Ibrutinib	2.7	DrugBanK
Dasatinib	4	DrugBanK
Imatinib	5	DrugBanK
Lapatinib	1	DrugBanK
Nilotinib	2.61	[144]
Pazopanib	1	DrugBanK
Ponatinib	1	DrugBanK
Regorafenib	0.5	DrugBanK
Sorafenib	0.5	DrugBanK
Sunitinib	5	DrugBanK
Trametinib	2.6	DrugBanK
Vandetanib	6.33	[145]

Table S6. The physicochemical properties of tyrosine kinase inhibitors.

Drug	Molecular weight (g/mol)	Water solubility (mg/L)	log*P*	pKa (acid)	pKa (base)
Afatinib	486	12.8	3.77	12.5	8.81
Axitinib	386	0.551	4.15	13.0	4.59
Bosutinib	530	9.50	4.09	15.5	8.43
Cabozantinib	502	1.99	4.01	13.5	5.90
Crizotinib	450	6.11	1.83	-	10.1
Dabrafenib	510	3.27	5.44	7.16	2.91
Dasatinib	488	12.8	5.06	11.0	3.10
Erlotinib	393	8.91	2.70	16.1	4.62
Gefitinib	447	27.0	3.20	16.1	5.40
Ibrutinib	441	3.00	2.76	-	3.74
Imatinib	494	14.6	4.38	12.5	8.27
Lapatinib	581	22.3	4.64	16.0	7.20
Nilotinib	530	2.01	4.51	12.4	5.92
Pazopanib	438	43.3	3.55	10.4	2.10
Ponatinib	533	2.95	3.94	11.4	2.77
Regorafenib	483	1.02	3.92	10.5	2.02
Sorafenib	465	1.71	3.80	11.6	2.03
Sunitinib	398	30.8	5.20	11.5	9.04
Trametinib	615	30.7	4.99	12.6	-
Vandetanib	475	10.2	4.54	13.8	9.13
Vemurafenib	490	0.362	4.62	7.17	3.20

Table S7. Tissue partition coefficients of tyrosine kinase inhibitors.

Drug	$K_{p\_observation}$	Reference	$K_{p\_prediction}$
Afatinib	1.77	[145]	2.41
Axitinib	-	-	1.76
Bosutinib	-	-	2.06
Cabozantinib	0.50	[146]	0.64
Crizotinib	0.55	[147]	0.41
Dabrafenib	-	-	1.98
Dasatinib	3.16	[148]	2.85
Erlotinib	0.23	[149]	0.75
Gefitinib	0.95	[150]	1.09
Ibrutinib	-	-	0.639
Imatinib	3.62	[151]	3.39
Lapatinib	0.12	[152]	2.74
Nilotinib	-	-	2.87
Pazopanib	-	-	0.306
Ponatinib	5.36	[153]	1.39
Regorafenib	0.56	[154]	0.95
Sorafenib	0.77	[155]	0.67
Sunitinib	2.20	[156]	3.85
Trametinib	3.26	[157]	3.66
Vandetanib	-	-	3.6
Vemurafenib	0.78	[158]	0.53

The $K_{p\_observation}$ represents data obtained from literature; $K_{p\_prediction}$ represents data obtained from the Poulin and Theil method [157].

Table S8. Summary literature for $IC_{50,3D}$ and $IC_{50,2D}$ of tyrosine kinase inhibitors.

Drug	$IC_{50,3D}$ (μM)	Reference	$IC_{50,2D}$ (μM)	Reference
Afatinib	-	-	9.72	
Axitinib	-	-	>100	
Bosutinib	-	-	10.2	
Cabozantinib	-	-	13.9	
Crizotinib	-	-	7.36	[160]
Dabrafenib	-	-	34.3	
Dasatinib	^{a}25	[161]	44.6	
Erlotinib	-	-	5.58	
Gefitinib	-	-	17.5	
Ibrutinib	-	-	10.6	
Imatinib	^{b}33	[162]	^{b}78.2	[162]
Lapatinib	-	-	108	
Nilotinib	^{a}29	[161]	8.31	
Pazopanib	-	-	5.09	[160]
Ponatinib	-	-	4.66	
Regorafenib	^{a}8.00	[161]	5.85	
Sorafenib	^{a}8.72	[163]	5.67	
Sunitinib	^{c}16.24		^{c}3.8	[162]
Trametinib	-	-	16.9	
Vandetanib	^{a}11.0	[161]	16.8	(160)
Vemurafenib	-	-	25.1	

$^{a}IC_{50}$ data for cellular viability (ATP depletion). $^{b}EC_{50}$ for tested compounds using peak count of Ca^{2+} as the readout. $^{c}IC_{50}$ data for mitochondrial membrane potential.

Reference

1. Sang L, Zhou Z, Luo S, Zhang Y, Qian H, Zhou Y, et al. An In Silico Platform to Predict Cardiotoxicity Risk of Anti-tumor Drug Combination with hiPSC-CMs Based In Vitro Study. Pharm Res. 2023.
2. Sang L, Yuan Y, Zhou Y, Zhou Z, Jiang M, Liu X, et al. A quantitative systems pharmacology approach to predict the safe-equivalent dose of doxorubicin in patients with cardiovascular comorbidity. CPT Pharmacometrics Syst Pharmacol. 2021;10(12):1512-24.
3. Freiwald M, Schmid U Fau - Fleury A, Fleury A Fau - Wind S, Wind S Fau - Stopfer P, Stopfer P Fau - Staab A, Staab A. Population pharmacokinetics of afatinib, an irreversible ErbB family blocker, in patients with various solid tumors. Cancer Chemother Pharmacol. 2014;73(4):759-70.
4. Garrett M, Poland B Fau - Brennan M, Brennan M Fau - Hee B, Hee B Fau - Pithavala YK, Pithavala Yk Fau - Amantea MA, Amantea MA. Population pharmacokinetic analysis of axitinib in healthy volunteers. Br J Clin Pharmacol. 2014;77(3):480-92.
5. Pfizer. 203341Clinical Pharmacology Review – Bosutinib. The U.S. Food and Drug Administration; 2012. Available at: https://www.accessdata.fda.gov/drugsatfda_docs/nda/2012/203341Orig1s000ClinPharmR.pdf (accessed on 18 June 2012).
6. Exelixis. NDA 203756 Clinical Pharmacology Review - Cabozantinib. The U.S. Food and Drug Administration; 2012. Available at: https://www.accessdata.fda.gov/drugsatfda_docs/nda/2012/203756Orig1s000ClinPharmR.pdf (accessed on 29 June 2012).
7. Pfizer. NDA 202570 Clinical Pharmacology Review - Crizotinib. The U.S. Food and Drug Administration; 2011. Available at: https://www.accessdata.fda.gov/drugsatfda_docs/nda/2011/202570Orig1s000ClinPharmR.pdf (accessed on 5 October 2011).
8. Novartis. NDA 217514 Clinical Inspection Summary - dabrafenib. The U.S. Food and Drug Administration; 2023. Available at: https://www.accessdata.fda.gov/drugsatfda_docs/nda/2023/217514Orig1s000OtherR.pdf (accessed on 24 May 2023).
9. He S, Zhao J, Bian J, Zhao Y, Li Y, Guo N, et al. Population Pharmacokinetics and Pharmacogenetics Analyses of Dasatinib in Chinese Patients with Chronic Myeloid Leukemia. Pharm Res. 2023;40(10):213-2422.
10. Evelina C, Guidi M, Khoudour N, Pascaline B-R, Fabre E, Tlemsani C, et al. Population Pharmacokinetics of Erlotinib in Patients With Non-small Cell Lung Cancer: Its Application for Individualized Dosing Regimens in Older Patients. Clin Ther. 2020;42(7):1302-16.
11. Li J, Karlsson Mo Fau - Brahmer J, Brahmer J Fau - Spitz A, Spitz A Fau - Zhao M, Zhao M Fau - Hidalgo M, Hidalgo M Fau - Baker SD, et al. CYP3A phenotyping approach to predict systemic exposure to EGFR tyrosine kinase inhibitors. J Natl Cancer Inst. 2006;98(23):1714-23.
12. Al-Ghazawi MA-O, Saleh MI, Najib O, Salem I, Najib N. Population Pharmacokinetics of Ibrutinib in Healthy Adults. Eur J Drug Metab Pharmacokinet. 2021;46(3):405-13.
13. Petain A, Kattygnarath D Fau - Azard J, Azard J Fau - Chatelut E, Chatelut E Fau - Delbaldo C, Delbaldo C Fau - Geoerger B, Geoerger B Fau - Barrois M, et al. Population

pharmacokinetics and pharmacogenetics of imatinib in children and adults. Clin Cancer Res. 2008;14(21):7102-9.

14. Thiessen B, Stewart C Fau - Tsao M, Tsao M Fau - Kamel-Reid S, Kamel-Reid S Fau - Schaiquevich P, Schaiquevich P Fau - Mason W, Mason W Fau - Easaw J, et al. A phase I/II trial of GW572016 (lapatinib) in recurrent glioblastoma multiforme: clinical outcomes, pharmacokinetics and molecular correlation. Cancer Chemother Pharmacol. 2010;65(2):353-61.
15. Giles FJ, Yin Oq Fau - Sallas WM, Sallas Wm Fau - le Coutre PD, le Coutre Pd Fau - Woodman RC, Woodman Rc Fau - Ottmann OG, Ottmann Og Fau - Baccarani M, et al. Nilotinib population pharmacokinetics and exposure-response analysis in patients with imatinib-resistant or -intolerant chronic myeloid leukemia. Eur J Clin Pharmacol. 2013;69(4):813-23.
16. Ozbey AA-O, Combarel D, Poinsignon V, Lovera C, Saada E, Mir OA, et al. Population Pharmacokinetic Analysis of Pazopanib in Patients and Determination of Target AUC. LID Pharmaceuticals (Basel). 2021;14(9):927.
17. Hanley MA-O, Diderichsen PM, Narasimhan N, Srivastava S, Gupta N, Venkatakrishnan K. Population Pharmacokinetics of Ponatinib in Healthy Adult Volunteers and Patients With Hematologic Malignancies and Model-Informed Dose Selection for Pediatric Development. J Clin Pharmacol. 2022;62(4):555-67.
18. Keunecke A, Hoefman S, Drenth HJ, Zisowsky J, Cleton A, Ploeger BA. Population pharmacokinetics of regorafenib in solid tumours: Exposure in clinical practice considering enterohepatic circulation and food intake. Br J Clin Pharmacol. 2020;86(12):2362-76.
19. Jain L, Woo S Fau - Gardner ER, Gardner Er Fau - Dahut WL, Dahut Wl Fau - Kohn EC, Kohn Ec Fau - Kummar S, Kummar S Fau - Mould DR, et al. Population pharmacokinetic analysis of sorafenib in patients with solid tumours. Br J Clin Pharmacol. 2011;72(2):294-305.
20. Wang EA-O, DuBois SG, Wetmore C, Khosravan R. Population pharmacokinetics-pharmacodynamics of sunitinib in pediatric patients with solid tumors. Cancer Chemother Pharmacol. 2020;86(2):181-92.
21. Balakirouchenane DA-O, Guégan SA-O, Csajka C, Jouinot A, Heidelberger V, Puszkiel AA-O, et al. Population Pharmacokinetics/Pharmacodynamics of Dabrafenib Plus Trametinib in Patients with BRAF-Mutated Metastatic Melanoma. Cancers (Basel). 2020;12(4):931.
22. Zhang L, Li S Fau - Zhang Y, Zhang Y Fau - Zhan J, Zhan J Fau - Zou B-Y, Zou By Fau - Smith R, Smith R Fau - Martin PD, et al. Pharmacokinetics and tolerability of vandetanib in Chinese patients with solid, malignant tumors: an open-label, phase I, rising multiple-dose study. Clin Ther. 2011;33(3):315-27.
23. Roche. NDA 202-429 BIOPHARMACEUTICS REVIEW– Vemurafenib. The U.S. Food and Drug Administration; 2011. Available at: https://www.accessdata.fda.gov/drugsatfda_docs/nda/2011/202429Orig1s000ClinPharmR.pdf (accessed on 31 May 2011).
24. Sendur MA, Fau. AS, Altundag K. Cardiotoxicity of novel HER2-targeted therapies. Curr Med Res Opin. 2013;29(8):1015-24.
25. Eskens FA, Fau. MC, Fau. PA, Fau. GJ, Fau. AA, Fau. HH, et al. A phase I dose escalation study of BIBW 2992, an irreversible dual inhibitor of epidermal growth factor receptor 1 (EGFR) and 2 (HER2) tyrosine kinase in a 2-week on, 2-week off schedule in patients with advanced solid tumours. Br J Cancer. 2008;98(1):80-5.

26. BMS. SPRYCEL™ (dasatinib). 2006. Available at: https://www.accessdata.fda.gov/drugsatfda_docs/label/2006/021986lbl.pdf (accessed on 19 February 2006).
27. Zamorano Jl Fau - Lancellotti P, Lancellotti P Fau - Rodriguez Muñoz D, Rodriguez Muñoz D Fau - Aboyans V, Aboyans V Fau - Asteggiano R, Asteggiano R Fau - Galderisi M, Galderisi M Fau - Habib G, et al. 2016 ESC Position Paper on cancer treatments and cardiovascular toxicity developed under the auspices of the ESC Committee for Practice Guidelines: The Task Force for cancer treatments and cardiovascular toxicity of the European Society of Cardiology (ESC). Eur Heart J. 2016;37(36):2768-801.
28. Atallah E, Durand Jb Fau - Kantarjian H, Kantarjian H Fau - Cortes J, Cortes J. Congestive heart failure is a rare event in patients receiving imatinib therapy. Blood. 2007;110(4):1223-7.
29. Hatfield A Fau - Owen S, Owen S Fau - Pilot PR, Pilot PR. In reply to 'Cardiotoxicity of the cancer therapeutic agent imatinib mesylate'. Nat Med. 2007;13(1):15-6.
30. Perez EA, Koehler M Fau - Byrne J, Byrne J Fau - Preston AJ, Preston Aj Fau - Rappold E, Rappold E Fau - Ewer MS, Ewer MS. Cardiac safety of lapatinib: pooled analysis of 3689 patients enrolled in clinical trials. Mayo Clin Proc. 2008;83(6):679-86.
31. Piccart-Gebhart M, Holmes E, Baselga J, de Azambuja E, Dueck AC, Viale G, et al. Adjuvant Lapatinib and Trastuzumab for Early Human Epidermal Growth Factor Receptor 2-Positive Breast Cancer: Results From the Randomized Phase III Adjuvant Lapatinib and/or Trastuzumab Treatment Optimization Trial. J Clin Oncol. 2016;34(10):1034-42.
32. Price KE, Saleem N Fau - Lee G, Lee G Fau - Steinberg M, Steinberg M. Potential of ponatinib to treat chronic myeloid leukemia and acute lymphoblastic leukemia. Onco Targets Ther. 2013;6:1111-8.
33. Motzer RJ, Hutson Te Fau - Cella D, Cella D Fau - Reeves J, Reeves J Fau - Hawkins R, Hawkins R Fau - Guo J, Guo J Fau - Nathan P, et al. Pazopanib versus sunitinib in metastatic renal-cell carcinoma. N Engl J Med. 2013;369(8):722-31.
34. Chen J, Wang J. Risk of regorafenib-induced cardiovascular events in patients with solid tumors: A systematic review and meta-analysis. Medicine (Baltimore). 2018;97(41):e12705.
35. Haas NB, Manola J, Ky B, Flaherty KT, Uzzo RG, Kane CJ, et al. Effects of Adjuvant Sorafenib and Sunitinib on Cardiac Function in Renal Cell Carcinoma Patients without Overt Metastases: Results from ASSURE, ECOG 2805. Clin Cancer Res. 2015;21(18):4048-54.
36. Khakoo AY, Kassiotis Cm Fau - Tannir N, Tannir N Fau - Plana JC, Plana Jc Fau - Halushka M, Halushka M Fau - Bickford C, Bickford C Fau - Trent J, 2nd, et al. Heart failure associated with sunitinib malate: a multitargeted receptor tyrosine kinase inhibitor. Cancer. 2008;112(11):2500-8.
37. Pedersen S, Larsen KO, Christensen AH, Svane IA-O, Zerahn B, Ellebaek EA-O. Cardiotoxicity in metastatic melanoma patients treated with BRAF and MEK inhibitors in a real-world setting. Acta Oncol. 2022;61(1):45-51.
38. Banks M, Crowell K, Proctor A, Jensen BA. Cardiovascular Effects of the MEK Inhibitor, Trametinib: A Case Report, Literature Review, and Consideration of Mechanism. Cardiovasc Toxicol. 2017;17(4):487-93.
39. GlaxoSmithKline. MEKINIST (trametinib) tablets. The U.S. Food and Drug Administration; 2013. Available at: https://www.accessdata.fda.gov/drugsatfda_docs/label/2013/204114s000lbl.pdf (accessed on

May 2013).
40. AstraZeneca. Vandetanib Tablets. 2011. Available at: https://www.accessdata.fda.gov/drugsatfda_docs/label/2011/022405s000lbl.pdf (accessed on 23 March 2011).
41. Yap TA, Vidal L Fau - Adam J, Adam J Fau - Stephens P, Stephens P Fau - Spicer J, Spicer J Fau - Shaw H, Shaw H Fau - Ang J, et al. Phase I trial of the irreversible EGFR and HER2 kinase inhibitor BIBW 2992 in patients with advanced solid tumors. J Clin Oncol. 2010;28(25):3965-72.
42. Murakami H, Tamura T Fau - Takahashi T, Takahashi T Fau - Nokihara H, Nokihara H Fau - Naito T, Naito T Fau - Nakamura Y, Nakamura Y Fau - Nishio K, et al. Phase I study of continuous afatinib (BIBW 2992) in patients with advanced non-small cell lung cancer after prior chemotherapy/erlotinib/gefitinib (LUX-Lung 4). Cancer Chemother Pharmacol. 2012;69(4):891-9.
43. Marshall J, Hwang J Fau - Eskens FALM, Eskens Fa Fau - Burger H, Burger H Fau - Malik S, Malik S Fau - Uttenreuther-Fischer M, Uttenreuther-Fischer M Fau - Stopfer P, et al. A Phase I, open-label, dose escalation study of afatinib, in a 3-week-on/1-week-off schedule in patients with advanced solid tumors. Invest New Drugs. 2013;31(2):399-408.
44. Wind S, Schmid M Fau - Erhardt J, Erhardt J Fau - Goeldner R-G, Goeldner Rg Fau - Stopfer P, Stopfer P. Pharmacokinetics of afatinib, a selective irreversible ErbB family blocker, in patients with advanced solid tumours. Clin Pharmacokinet. 2013;52(12).
45. Wind S, Giessmann T Fau - Jungnik A, Jungnik A Fau - Brand T, Brand T Fau - Marzin K, Marzin K Fau - Bertulis J, Bertulis J Fau - Hocke J, et al. Pharmacokinetic drug interactions of afatinib with rifampicin and ritonavir. Clin Drug Investig. 2014;34(3):173-82.
46. Mukai H, Masuda N, Ishiguro H, Mitsuma A, Shibata T, Yamamura J, et al. Phase I trial of afatinib plus vinorelbine in Japanese patients with advanced solid tumors, including breast cancer. Cancer Chemother Pharmacol. 2015;76(4):739-50.
47. Hiret SA-O, Isambert N, Gomez-Roca C, Bennouna J, Sassi M, de Mont-Serrat H, et al. Phase I dose-escalation trial of afatinib, an irreversible ErbB family blocker, in combination with gemcitabine or docetaxel in patients with relapsed or refractory solid tumors. Invest New Drugs. 2018;36(6):1044-59.
48. Pithavala YK, Tortorici M Fau - Toh M, Toh M Fau - Garrett M, Garrett M Fau - Hee B, Hee B Fau - Kuruganti U, Kuruganti U Fau - Ni G, et al. Effect of rifampin on the pharmacokinetics of Axitinib (AG-013736) in Japanese and Caucasian healthy volunteers. Cancer Chemother Pharmacol. 2010;65(3):563-70.
49. Tortorici MA, Toh M Fau - Rahavendran SV, Rahavendran Sv Fau - Labadie RR, Labadie Rr Fau - Alvey CW, Alvey Cw Fau - Marbury T, Marbury T Fau - Fuentes E, et al. Influence of mild and moderate hepatic impairment on axitinib pharmacokinetics. Invest New Drugs. 2011;29(6):1370-80.
50. Pithavala YK, Tong W Fau - Mount J, Mount J Fau - Rahavendran SV, Rahavendran Sv Fau - Garrett M, Garrett M Fau - Hee B, Hee B Fau - Selaru P, et al. Effect of ketoconazole on the pharmacokinetics of axitinib in healthy volunteers. Invest New Drugs. 2012;30(1):273-81.
51. Pithavala YK, Chen Y Fau - Toh M, Toh M Fau - Selaru P, Selaru P Fau - LaBadie RR, LaBadie Rr Fau - Garrett M, Garrett M Fau - Hee B, et al. Evaluation of the effect of food on the pharmacokinetics of axitinib in healthy volunteers. Cancer Chemother Pharmacol.

2012;70(1):103-12.
52. Smith BJ, Pithavala Y Fau - Bu H-Z, Bu Hz Fau - Kang P, Kang P Fau - Hee B, Hee B Fau - Deese AJ, Deese Aj Fau - Pool WF, et al. Pharmacokinetics, metabolism, and excretion of [14C]axitinib, a vascular endothelial growth factor receptor tyrosine kinase inhibitor, in humans. Drug Metab Dispos. 2014;42(5):918-31.
53. Hsyu PH, Pignataro DS, Matschke K. Absolute Bioavailability of Bosutinib in Healthy Subjects From an Open-Label, Randomized, 2-Period Crossover Study. Clin Pharmacol Drug Dev. 2018;7(4):373-81.
54. Hsyu PH, Pignataro DS, Matschke K. Effect of aprepitant, a moderate CYP3A4 inhibitor, on bosutinib exposure in healthy subjects. Eur J Clin Pharmacol. 2017;73(1):49-56.
55. Abbas R Fau - Boni J, Boni J Fau - Sonnichsen D, Sonnichsen D. Effect of rifampin on the pharmacokinetics of bosutinib, a dual Src/Abl tyrosine kinase inhibitor, when administered concomitantly to healthy subjects. Drug Metab Pers Ther. 2015;30(1):57-63.
56. Abbas R, Hug Ba Fau - Leister C, Leister C Fau - Sonnichsen D, Sonnichsen D. A randomized, crossover, placebo- and moxifloxacin-controlled study to evaluate the effects of bosutinib (SKI-606), a dual Src/Abl tyrosine kinase inhibitor, on cardiac repolarization in healthy adult subjects. Int J Cancer. 2012;131(3):E304-11.
57. Lacy SA, Miles DR, Nguyen LT. Clinical Pharmacokinetics and Pharmacodynamics of Cabozantinib. Clin Pharmacokinet. 2017;56(5):477-91.
58. Nguyen L, Benrimoh N Fau - Xie Y, Xie Y Fau - Offman E, Offman E Fau - Lacy S, Lacy S. Pharmacokinetics of cabozantinib tablet and capsule formulations in healthy adults. Anticancer Drugs. 2016;27(7):669-78.
59. Nguyen L, Holland J, Mamelok R, Laberge MK, Grenier J, Swearingen D, et al. Evaluation of the effect of food and gastric pH on the single-dose pharmacokinetics of cabozantinib in healthy adult subjects. J Clin Pharmacol. 2015;55(11).
60. Xu H, O'Gorman M Fau - Boutros T, Boutros T Fau - Brega N, Brega N Fau - Kantaridis C, Kantaridis C Fau - Tan W, Tan W Fau - Bello A, et al. Evaluation of crizotinib absolute bioavailability, the bioequivalence of three oral formulations, and the effect of food on crizotinib pharmacokinetics in healthy subjects. J Clin Pharmacol. 2015;55(1):104-13.
61. Xu H, O'Gorman M, Tan W, Brega N, Bello A. The effects of ketoconazole and rifampin on the single-dose pharmacokinetics of crizotinib in healthy subjects. Eur J Clin Pharmacol. 2015;71(12):1441-9.
62. Clark JW, Camidge DR, Kwak EL, Maki RG, Shapiro GI, Chen I, et al. Dose-escalation trial of the ALK, MET & ROS1 inhibitor, crizotinib, in patients with advanced cancer. Future Oncol. 2020;16(1):4289-301.
63. Yamazaki S, Johnson TR, Smith BJ. Prediction of Drug-Drug Interactions with Crizotinib as the CYP3A Substrate Using a Physiologically Based Pharmacokinetic Model. Drug Metab Dispos. 2015;43(10):1417-29.
64. El-Khoueiry AA-O, Sarantopoulos J, O'Bryant CL, Ciombor KK, Xu H, O'Gorman M, et al. Evaluation of hepatic impairment on pharmacokinetics and safety of crizotinib in patients with advanced cancer. Cancer Chemother Pharmacol. 2018;81(4):659-70.
65. Christopher LJ, Cui D Fau - Wu C, Wu C Fau - Luo R, Luo R Fau - Manning JA, Manning Ja Fau - Bonacorsi SJ, Bonacorsi Sj Fau - Lago M, et al. Metabolism and disposition of dasatinib after oral administration to humans. Drug Metab Dispos. 2008;36(7):1357-64.

66. Furlong MT, Agrawal S Fau - Hawthorne D, Hawthorne D Fau - Lago M, Lago M Fau - Unger S, Unger S Fau - Krueger L, Krueger L Fau - Stouffer B, et al. A validated LC-MS/MS assay for the simultaneous determination of the anti-leukemic agent dasatinib and two pharmacologically active metabolites in human plasma: application to a clinical pharmacokinetic study. J Pharm Biomed Anal. 2012;58:130-5.
67. Takahashi S, Miyazaki M Fau - Okamoto I, Okamoto I Fau - Ito Y, Ito Y Fau - Ueda K, Ueda K Fau - Seriu T, Seriu T Fau - Nakagawa K, et al. Phase I study of dasatinib (BMS-354825) in Japanese patients with solid tumors. Cancer Sci. 2011;102(11):2058-64.
68. Araujo JC, Mathew P Fau - Armstrong AJ, Armstrong Aj Fau - Braud EL, Braud El Fau - Posadas E, Posadas E Fau - Lonberg M, Lonberg M Fau - Gallick GE, et al. Dasatinib combined with docetaxel for castration-resistant prostate cancer: results from a phase 1-2 study. Cancer. 2012;118(1):63-71.
69. Yago MR, Frymoyer A Fau - Benet LZ, Benet Lz Fau - Smelick GS, Smelick Gs Fau - Frassetto LA, Frassetto La Fau - Ding X, Ding X Fau - Dean B, et al. The use of betaine HCl to enhance dasatinib absorption in healthy volunteers with rabeprazole-induced hypochlorhydria. AAPS J. 2014;16(6):1358-65.
70. Kong J, Chen N Fau - Fu HX, Fu Hx Fau - Hang TJ, Hang Tj Fau - Song M, Song M Fau - Jiang H, Jiang H. Pharmacokinetics of generic dasatinib in the management of chronic myeloid leukemia in the choronie phase. Zhonghua Xue Ye Xue Za Zhi. 2016;37(11):957-60.
71. Denton CL, Minthorn E Fau - Carson SW, Carson Sw Fau - Young GC, Young Gc Fau - Richards-Peterson LE, Richards-Peterson Le Fau - Botbyl J, Botbyl J Fau - Han C, et al. Concomitant oral and intravenous pharmacokinetics of dabrafenib, a BRAF inhibitor, in patients with BRAF V600 mutation-positive solid tumors. J Clin Pharmacol. 2013;53(9):955-61.
72. Ouellet D, Grossmann Kf Fau - Limentani G, Limentani G Fau - Nebot N, Nebot N Fau - Lan K, Lan K Fau - Knowles L, Knowles L Fau - Gordon MS, et al. Effects of particle size, food, and capsule shell composition on the oral bioavailability of dabrafenib, a BRAF inhibitor, in patients with BRAF mutation-positive tumors. J Pharm Sci. 2013;102(9):3100-9.
73. Falchook GS, Long GV, Kurzrock R, Kim KB, Arkenau HT, Brown MP, et al. Dose selection, pharmacokinetics, and pharmacodynamics of BRAF inhibitor dabrafenib (GSK2118436). Clin Cancer Res. 2014;20(17):4449-58.
74. O'Bryant CL, Haluska P Fau - Rosen L, Rosen L Fau - Ramanathan RK, Ramanathan Rk Fau - Venugopal B, Venugopal B Fau - Leong S, Leong S Fau - Boinpally R, et al. An open-label study to describe pharmacokinetic parameters of erlotinib in patients with advanced solid tumors with adequate and moderately impaired hepatic function. Cancer Chemother Pharmacol. 2012;69(3):605-12.
75. Hamilton M, Wolf Jl Fau - Rusk J, Rusk J Fau - Beard SE, Beard Se Fau - Clark GM, Clark Gm Fau - Witt K, Witt K Fau - Cagnoni PJ, et al. Effects of smoking on the pharmacokinetics of erlotinib. Clin Cancer Res. 2006;12(7 Pt 1):2166-71.
76. Das M, Padda SK, Frymoyer A, Zhou L, Riess JW, Neal JW, et al. Dovitinib and erlotinib in patients with metastatic non-small cell lung cancer: A drug-drug interaction. Lung Cancer. 2015;89(3):280-6.
77. Wang L, Ruan Z, Yang D, Hu Y, Liang J, Chen J, et al. Pharmacokinetics and Bioequivalence Evaluation of Erlotinib Hydrochloride Tablets: Randomized, Open-Label, 2-Period Crossover

Study in Healthy Chinese Subjects. Clin Pharmacol Drug Dev. 2021;10(2):166-72.
78. Kletzl H, Giraudon M Fau - Ducray PS, Ducray Ps Fau - Abt M, Abt M Fau - Hamilton M, Hamilton M Fau - Lum BL, Lum BL. Effect of gastric pH on erlotinib pharmacokinetics in healthy individuals: omeprazole and ranitidine. Anticancer Drugs. 2015;26(5):565-72.
79. Masago K, Togashi Y Fau - Fukudo M, Fukudo M Fau - Terada T, Terada T Fau - Irisa K, Irisa K Fau - Sakamori Y, Sakamori Y Fau - Kim YH, et al. Plasma and pleural fluid pharmacokinetics of erlotinib and its active metabolite OSI-420 in patients with non-small-cell lung cancer with pleural effusion. Clin Lung Cancer. 2011;12(5):307-12.
80. Choi HG, Jeon Jy Fau - Im Y-J, Im Yj Fau - Kim Y, Kim Y Fau - Song E-K, Song Ek Fau - Seo Y-H, Seo Yh Fau - Cho S-J, et al. Pharmacokinetic properties of two erlotinib 150 mg formulations with a genetic effect evaluation in healthy Korean subjects. Clin Drug Investig. 2015;35(1):31-43.
81. Hughes AN, O'Brien Me Fau - Petty WJ, Petty Wj Fau - Chick JB, Chick Jb Fau - Rankin E, Rankin E Fau - Woll PJ, Woll Pj Fau - Dunlop D, et al. Overcoming CYP1A1/1A2 mediated induction of metabolism by escalating erlotinib dose in current smokers. J Clin Oncol. 2009;27(8):1220-6.
82. Yamamoto N, Horiike A Fau - Fujisaka Y, Fujisaka Y Fau - Murakami H, Murakami H Fau - Shimoyama T, Shimoyama T Fau - Yamada Y, Yamada Y Fau - Tamura T, et al. Phase I dose-finding and pharmacokinetic study of the oral epidermal growth factor receptor tyrosine kinase inhibitor Ro50-8231 (erlotinib) in Japanese patients with solid tumors. Cancer Chemother Pharmacol. 2008;61(3):489-96.
83. Ling J, Fettner S Fau - Lum BL, Lum Bl Fau - Riek M, Riek M Fau - Rakhit A, Rakhit A. Effect of food on the pharmacokinetics of erlotinib, an orally active epidermal growth factor receptor tyrosine-kinase inhibitor, in healthy individuals. Anticancer Drugs. 2008;19(2):209-16.
84. Cantarini MV, McFarquhar T Fau - Smith RP, Smith Rp Fau - Bailey C, Bailey C Fau - Marshall AL, Marshall AL. Relative bioavailability and safety profile of gefitinib administered as a tablet or as a dispersion preparation via drink or nasogastric tube: results of a randomized, open-label, three-period crossover study in healthy volunteers. Clin Ther. 2004;26(10):1630-6.
85. Cantarini MV, Macpherson Mp Fau - Marshall AL, Marshall Al Fau - Robinson AV, Robinson Av Fau - Bailey CJ, Bailey CJ. A phase I study to determine the effect of tamoxifen on the pharmacokinetics of a single 250 mg oral dose of gefitinib (IRESSA) in healthy male volunteers. Cancer Chemother Pharmacol. 2005;56(6):557-62.
86. Swaisland HC, Ranson M Fau - Smith RP, Smith Rp Fau - Leadbetter J, Leadbetter J Fau - Laight A, Laight A Fau - McKillop D, McKillop D Fau - Wild MJ, et al. Pharmacokinetic drug interactions of gefitinib with rifampicin, itraconazole and metoprolol. Clin Pharmacokinet. 2005;44(10):1067-81.
87. Adjei AA, Molina Jr Fau - Mandrekar SJ, Mandrekar Sj Fau - Marks R, Marks R Fau - Reid JR, Reid Jr Fau - Croghan G, Croghan G Fau - Hanson LJ, et al. Phase I trial of sorafenib in combination with gefitinib in patients with refractory or recurrent non-small cell lung cancer. Clin Cancer Res. 2007;13(9):2684-91.
88. Bergman E, Forsell P Fau - Persson EM, Persson Em Fau - Knutson L, Knutson L Fau - Dickinson P, Dickinson P Fau - Smith R, Smith R Fau - Swaisland H, et al. Pharmacokinetics

of gefitinib in humans: the influence of gastrointestinal factors. Int J Pharm. 2007;341(1-2):134-42.

89. Cantarini MV, Bailey Cj Fau - Collins B, Collins B Fau - Smith RP, Smith RP. The relative bioavailability of gefitinib administered by granular formulation. Cancer Chemother Pharmacol. 2008;62(2):203-8.
90. Tang W, Tomkinson H, Masson E. Effect of Sustained Elevated Gastric pH Levels on Gefitinib Exposure. Clin Pharmacol Drug Dev. 2017;6(5):517-23.
91. de Jong J, Hellemans P, De Wilde S, Patricia D, Masterson T, Manikhas G, et al. A drug-drug interaction study of ibrutinib with moderate/strong CYP3A inhibitors in patients with B-cell malignancies. Leuk Lymphoma. 2018;59(12):2888-95.
92. de Vries R, Smit JW, Hellemans P, Jiao J, Murphy J, Skee D, et al. Stable isotope-labelled intravenous microdose for absolute bioavailability and effect of grapefruit juice on ibrutinib in healthy adults. Br J Clin Pharmacol. 2016;81(2):235-45.
93. Younes A, Thieblemont C, Morschhauser F, Flinn I, Friedberg JW, Amorim S, et al. Combination of ibrutinib with rituximab, cyclophosphamide, doxorubicin, vincristine, and prednisone (R-CHOP) for treatment-naive patients with CD20-positive B-cell non-Hodgkin lymphoma: a non-randomised, phase 1b study. Lancet Oncol. 2014;15(9):1019-26.
94. Advani RH, Buggy Jj Fau - Sharman JP, Sharman Jp Fau - Smith SM, Smith Sm Fau - Boyd TE, Boyd Te Fau - Grant B, Grant B Fau - Kolibaba KS, et al. Bruton tyrosine kinase inhibitor ibrutinib (PCI-32765) has significant activity in patients with relapsed/refractory B-cell malignancies. J Clin Oncol. 2013;31(1):88-94.
95. Nikolova Z, Peng B Fau - Hubert M, Hubert M Fau - Sieberling M, Sieberling M Fau - Keller U, Keller U Fau - Ho Y-Y, Ho Yy Fau - Schran H, et al. Bioequivalence, safety, and tolerability of imatinib tablets compared with capsules. Cancer Chemother Pharmacol. 2004;53(5):433-8.
96. Peng B, Hayes M Fau - Resta D, Resta D Fau - Racine-Poon A, Racine-Poon A Fau - Druker BJ, Druker Bj Fau - Talpaz M, Talpaz M Fau - Sawyers CL, et al. Pharmacokinetics and pharmacodynamics of imatinib in a phase I trial with chronic myeloid leukemia patients. J Clin Oncol. 2004;22(5):935-42.
97. Bolton AE, Peng B Fau - Hubert M, Hubert M Fau - Krebs-Brown A, Krebs-Brown A Fau - Capdeville R, Capdeville R Fau - Keller U, Keller U Fau - Seiberling M, et al. Effect of rifampicin on the pharmacokinetics of imatinib mesylate (Gleevec, STI571) in healthy subjects. Cancer Chemother Pharmacol. 2004;53(2):102-6.
98. Roosendaal JA-O, Groenland SL, Rosing H, Lucas L, Venekamp N, Nuijen B, et al. Determination of the absolute bioavailability of oral imatinib using a stable isotopically labeled intravenous imatinib-d8 microdose. Eur J Clin Pharmacol. 2020;76(8):1075-82.
99. Arora R, Sharma M, Monif T, Iyer S. A Multi-centric Bioequivalence Trial in Ph+ Chronic Myeloid Leukemia Patients to Assess Bioequivalence and Safety Evaluation of Generic Imatinib Mesylate 400 mg Tablets. Cancer Res Treat. 2016;48(3):1120-9.
100. Frye RF, Fitzgerald Sm Fau - Lagattuta TF, Lagattuta Tf Fau - Hruska MW, Hruska Mw Fau - Egorin MJ, Egorin MJ. Effect of St John's wort on imatinib mesylate pharmacokinetics. Clin Pharmacol Ther. 2004;76(4):323-9.
101. Gadgeel SM, Lew Dl Fau - Synold TW, Synold Tw Fau - LoRusso P, LoRusso P Fau - Chung V, Chung V Fau - Christensen SD, Christensen Sd Fau - Smith DC, et al. Phase I study evaluating the combination of lapatinib (a Her2/Neu and EGFR inhibitor) and everolimus (an

mTOR inhibitor) in patients with advanced cancers: South West Oncology Group (SWOG) Study S0528. Cancer Chemother Pharmacol. 2013;72(5):1089-96.

102. Simonelli M, Zucali Pa Fau - Lorenzi E, Lorenzi E Fau - Rubino L, Rubino L Fau - De Vincenzo F, De Vincenzo F Fau - De Sanctis R, De Sanctis R Fau - Perrino M, et al. Phase I pharmacokinetic and pharmacodynamic study of lapatinib in combination with sorafenib in patients with advanced refractory solid tumors. Eur J Cancer. 2013;49(5):989-98.
103. Devriese LA, Koch Km Fau - Mergui-Roelvink M, Mergui-Roelvink M Fau - Matthys GM, Matthys Gm Fau - Ma WW, Ma Ww Fau - Robidoux A, Robidoux A Fau - Stephenson JJ, et al. Effects of low-fat and high-fat meals on steady-state pharmacokinetics of lapatinib in patients with advanced solid tumours. Invest New Drugs. 2014;32(3):481-8.
104. Koch KM, Im YH, Kim SB, Urruticoechea Ribate A, Stephenson J, Botbyl J, et al. Effects of Esomeprazole on the Pharmacokinetics of Lapatinib in Breast Cancer Patients. Clin Pharmacol Drug Dev. 2013;2(4):336-41.
105. Tanaka C, Yin Oq Fau - Sethuraman V, Sethuraman V Fau - Smith T, Smith T Fau - Wang X, Wang X Fau - Grouss K, Grouss K Fau - Kantarjian H, et al. Clinical pharmacokinetics of the BCR-ABL tyrosine kinase inhibitor nilotinib. Clin Pharmacol Ther. 2010;87(2):197-203.
106. Trent J, Molimard M. Pharmacokinetics and pharmacodynamics of nilotinib in gastrointestinal stromal tumors. Semin Oncol. 2011;38(Suppl 1):S28-S33.
107. Zhou L, Meng F Fau - Yin O, Yin O Fau - Wang J, Wang J Fau - Wang Y, Wang Y Fau - Wei Y, Wei Y Fau - Hu P, et al. Nilotinib for imatinib-resistant or -intolerant chronic myeloid leukemia in chronic phase, accelerated phase, or blast crisis: a single- and multiple-dose, open-label pharmacokinetic study in Chinese patients. Clin Ther. 2009;31(7):1568-75.
108. Tawbi HA, Tran Al Fau - Christner SM, Christner Sm Fau - Lin Y, Lin Y Fau - Johnson M, Johnson M Fau - Mowrey E, Mowrey E Fau - Appleman LR, et al. Calcium carbonate does not affect nilotinib pharmacokinetics in healthy volunteers. Cancer Chemother Pharmacol. 2013;72(5):1143-7.
109. Wagner C, Kesisoglou F, Pepin XJH, Parrott N, Emami Riedmaier A. Use of Physiologically Based Pharmacokinetic Modeling for Predicting Drug-Food Interactions: Recommendations for Improving Predictive Performance of Low Confidence Food Effect Models. AAPS J. 2021;23(4):85.
110. Shibata SI, Chung V Fau - Synold TW, Synold Tw Fau - Longmate JA, Longmate Ja Fau - Suttle AB, Suttle Ab Fau - Ottesen LH, Ottesen Lh Fau - Lenz H-J, et al. Phase I study of pazopanib in patients with advanced solid tumors and hepatic dysfunction: a National Cancer Institute Organ Dysfunction Working Group study. Clin Cancer Res. 2013;19(13):3631-9.
111. Lubberman FJE, Gelderblom H, Hamberg P, Vervenne WL, Mulder SF, Jansman FA-O, et al. The Effect of Using Pazopanib With Food vs. Fasted on Pharmacokinetics, Patient Safety, and Preference (DIET Study). Clin Pharmacol Ther. 2019;106(5):1076-82.
112. Kerklaan BM, Lolkema MP, Devriese LA, Voest EE, Nol-Boekel A, Mergui-Roelvink M, et al. Phase I and pharmacological study of pazopanib in combination with oral topotecan in patients with advanced solid tumours. Br J Cancer. 2015;113(5):706-15.
113. Narasimhan NI, Dorer DJ, Davis J, Turner CD, Sonnichsen D. Evaluation of the effect of multiple doses of rifampin on the pharmacokinetics and safety of ponatinib in healthy subjects. Clin Pharmacol Drug Dev. 2015;4(5):354-60.
114. Narasimhan NI, Dorer Dj Fau - Davis J, Davis J Fau - Turner CD, Turner Cd Fau - Sonnichsen

D, Sonnichsen D. Evaluation of the effect of multiple doses of lansoprazole on the pharmacokinetics and safety of ponatinib in healthy subjects. Clin Drug Investig. 2014;34(10):723-9.
115. Narasimhan NI, Dorer Dj Fau - Niland K, Niland K Fau - Haluska F, Haluska F Fau - Sonnichsen D, Sonnichsen D. Effects of food on the pharmacokinetics of ponatinib in healthy subjects. J Clin Pharm Ther. 2013;38(6):440-4.
116. Ye YE, Woodward CN, Narasimhan NI. Absorption, metabolism, and excretion of [14C]ponatinib after a single oral dose in humans. Cancer Chemother Pharmacol. 2017;79(3):507-18.
117. Zhang Q, Wang ZA-OX, Wu J, Zhou Z, Zhou R, Hu WA-O. Bioequivalence and Pharmacokinetic Evaluation of Two Oral Formulations of Regorafenib: An Open-Label, Randomised, Single-Dose, Two-Period, Two-Way Crossover Clinical Trial in Healthy Chinese Volunteers Under Fasting and Fed Conditions. Drug Des Devel Ther. 2021;15:3277-88.
118. Mross K, Frost A Fau - Steinbild S, Steinbild S Fau - Hedbom S, Hedbom S Fau - Büchert M, Büchert M Fau - Fasol U, Fasol U Fau - Unger C, et al. A phase I dose-escalation study of regorafenib (BAY 73-4506), an inhibitor of oncogenic, angiogenic, and stromal kinases, in patients with advanced solid tumors. Clin Cancer Res. 2012;18(9):2658-67.
119. Strumberg D, Scheulen Me Fau - Schultheis B, Schultheis B Fau - Richly H, Richly H Fau - Frost A, Frost A Fau - Büchert M, Büchert M Fau - Christensen O, et al. Regorafenib (BAY 73-4506) in advanced colorectal cancer: a phase I study. Br J Cancer. 2012;106(11):1722-7.
120. Kubota Y, Fujita KI, Takahashi T, Sunakawa Y, Ishida H, Hamada K, et al. Higher Systemic Exposure to Unbound Active Metabolites of Regorafenib Is Associated With Short Progression-Free Survival in Colorectal Cancer Patients. Clin Pharmacol Ther. 2020;108(3):586-95.
121. Awada A, Hendlisz A Fau - Gil T, Gil T Fau - Bartholomeus S, Bartholomeus S Fau - Mano M, Mano M Fau - de Valeriola D, de Valeriola D Fau - Strumberg D, et al. Phase I safety and pharmacokinetics of BAY 43-9006 administered for 21 days on/7 days off in patients with advanced, refractory solid tumours. Br J Cancer. 2005;92(10):1855-61.
122. Clark JW, Eder Jp Fau - Ryan D, Ryan D Fau - Lathia C, Lathia C Fau - Lenz H-J, Lenz HJ. Safety and pharmacokinetics of the dual action Raf kinase and vascular endothelial growth factor receptor inhibitor, BAY 43-9006, in patients with advanced, refractory solid tumors. Clin Cancer Res. 2005;11(15):5472-80.
123. Li J, Kluger H, Devine L, Lee JJ, Kelly WK, Rink L, et al. Phase I study of safety and tolerability of sunitinib in combination with sirolimus in patients with refractory solid malignancies and determination of VEGF (VEGF-A) and soluble VEGF-R2 (sVEGFR2) in plasma. Cancer Chemother Pharmacol. 2016;77(6):1193-200.
124. Leong S, Eckhardt Sg Fau - Chan E, Chan E Fau - Messersmith WA, Messersmith Wa Fau - Spratlin J, Spratlin J Fau - Camidge DR, Camidge Dr Fau - Diab S, et al. A phase I study of sunitinib combined with modified FOLFOX6 in patients with advanced solid tumors. Cancer Chemother Pharmacol. 2012;70(1):65-74.
125. Bello CL, Garrett M Fau - Sherman L, Sherman L Fau - Smeraglia J, Smeraglia J Fau - Ryan B, Ryan B Fau - Toh M, Toh M. Pharmacokinetics of sunitinib malate in subjects with hepatic impairment. Cancer Chemother Pharmacol. 2010;66(4):699-707.
126. Britten CD, Kabbinavar F Fau - Hecht JR, Hecht Jr Fau - Bello CL, Bello Cl Fau - Li J, Li J

Fau - Baum C, Baum C Fau - Slamon D, et al. A phase I and pharmacokinetic study of sunitinib administered daily for 2 weeks, followed by a 1-week off period. Cancer Chemother Pharmacol. 2008;61(3):515-24.

127. Shirao K, Nishida T Fau - Doi T, Doi T Fau - Komatsu Y, Komatsu Y Fau - Muro K, Muro K Fau - Li Y, Li Y Fau - Ueda E, et al. Phase I/II study of sunitinib malate in Japanese patients with gastrointestinal stromal tumor after failure of prior treatment with imatinib mesylate. Invest New Drugs. 2010;28(6):866-75.
128. Bello CL, Sherman L Fau - Zhou J, Zhou J Fau - Verkh L, Verkh L Fau - Smeraglia J, Smeraglia J Fau - Mount J, Mount J Fau - Klamerus KJ, et al. Effect of food on the pharmacokinetics of sunitinib malate (SU11248), a multi-targeted receptor tyrosine kinase inhibitor: results from a phase I study in healthy subjects. Anticancer Drugs. 2006;17(3):353-8.
129. Kasuga A, Nakagawa K, Nagashima F, Shimizu T, Naruge D, Nishina S, et al. A phase I/Ib study of trametinib (GSK1120212) alone and in combination with gemcitabine in Japanese patients with advanced solid tumors. Invest New Drugs. 2015;33(5):1058-67.
130. Cox DS, Papadopoulos K Fau - Fang L, Fang L Fau - Bauman J, Bauman J Fau - LoRusso P, LoRusso P Fau - Tolcher A, Tolcher A Fau - Patnaik A, et al. Evaluation of the effects of food on the single-dose pharmacokinetics of trametinib, a first-in-class MEK inhibitor, in patients with cancer. J Clin Pharmacol. 2013;53(9):946-54.
131. Cox DS, Allred A, Zhou Y, Infante JR, Gordon MS, Bendell J, et al. Relative bioavailability of pediatric oral solution and tablet formulations of trametinib in adult patients with solid tumors. Clin Pharmacol Drug Dev. 2015;4(4):287-94.
132. Leonowens C, Pendry C Fau - Bauman J, Bauman J Fau - Young GC, Young Gc Fau - Ho M, Ho M Fau - Henriquez F, Henriquez F Fau - Fang L, et al. Concomitant oral and intravenous pharmacokinetics of trametinib, a MEK inhibitor, in subjects with solid tumours. Br J Clin Pharmacol. 2014;78(3):524-32.
133. Infante JR, Fecher La Fau - Falchook GS, Falchook Gs Fau - Nallapareddy S, Nallapareddy S Fau - Gordon MS, Gordon Ms Fau - Becerra C, Becerra C Fau - DeMarini DJ, et al. Safety, pharmacokinetic, pharmacodynamic, and efficacy data for the oral MEK inhibitor trametinib: a phase 1 dose-escalation trial. Lancet Oncol. 2012;13(8):773-81.
134. Martin P, Oliver S Fau - Robertson J, Robertson J Fau - Kennedy S-J, Kennedy Sj Fau - Read J, Read J Fau - Duvauchelle T, Duvauchelle T. Pharmacokinetic drug interactions with vandetanib during coadministration with rifampicin or itraconazole. Drugs R D. 2011;11(1):37-51.
135. Martin P, Oliver S Fau - Kennedy S-J, Kennedy Sj Fau - Partridge E, Partridge E Fau - Hutchison M, Hutchison M Fau - Clarke D, Clarke D Fau - Giles P, et al. Pharmacokinetics of vandetanib: three phase I studies in healthy subjects. Clin Ther. 2012;34(1):221-37.
136. Johansson S, Read J Fau - Oliver S, Oliver S Fau - Steinberg M, Steinberg M Fau - Li Y, Li Y Fau - Lisbon E, Lisbon E Fau - Mathews D, et al. Pharmacokinetic evaluations of the co-administrations of vandetanib and metformin, digoxin, midazolam, omeprazole or ranitidine. Clin Pharmacokinet. 2014;53(9):837-47.
137. Holden SN, Eckhardt Sg Fau - Basser R, Basser R Fau - de Boer R, de Boer R Fau - Rischin D, Rischin D Fau - Green M, Green M Fau - Rosenthal MA, et al. Clinical evaluation of ZD6474, an orally active inhibitor of VEGF and EGF receptor signaling, in patients with solid, malignant tumors. Ann Oncol. 2005;16(8):1391-7.

138. Zhang W, Mathisen M, Goodman GR, Forbes H, Song Y, Bertran E, et al. Effect of Itraconazole, a Potent CYP3A4 Inhibitor, on the Steady-State Pharmacokinetics of Vemurafenib in Patients With BRAF(V600) Mutation-Positive Malignancies. Clin Pharmacol Drug Dev. 2021;10(1):39-45.
139. Zhang W, McIntyre C, Forbes H, Gaafar R, Kohail H, Beck JT, et al. Effect of Rifampicin on the Pharmacokinetics of a Single Dose of Vemurafenib in Patients With BRAF(V600) Mutation-Positive Metastatic Malignancy. Clin Pharmacol Drug Dev. 2019;8(6):837-43.
140. Chisholm JA-O, Suvada J, Dunkel IJ, Casanova M, Zhang W, Ritchie N, et al. BRIM-P: A phase I, open-label, multicenter, dose-escalation study of vemurafenib in pediatric patients with surgically incurable, BRAF mutation-positive melanoma. Pediatr Blood Cancer. 2018;65(5):e26947.
141. Kopetz S, Desai J, Chan E, Hecht JR, O'Dwyer PJ, Maru D, et al. Phase II Pilot Study of Vemurafenib in Patients With Metastatic BRAF-Mutated Colorectal Cancer. J Clin Oncol. 2015;33(34):4032-8.
142. Ribas A, Zhang W Fau - Chang I, Chang I Fau - Shirai K, Shirai K Fau - Ernstoff MS, Ernstoff Ms Fau - Daud A, Daud A Fau - Cowey CL, et al. The effects of a high-fat meal on single-dose vemurafenib pharmacokinetics. J Clin Pharmacol. 2014;54(4):368-74.
143. Kayesh R, Farasyn T, Crowe A, Liu Q, Pahwa S, Alam K, et al. Assessing OATP1B1- and OATP1B3-Mediated Drug-Drug Interaction Potential of Vemurafenib Using R-Value and Physiologically-Based Pharmacokinetic Models. J Pharm Sci. 2021;110(1):314-24.
144. Ando H, Yoshinaga T, Yamamoto W, Asakura K, Uda T, Taniguchi T, et al. A new paradigm for drug-induced torsadogenic risk assessment using human iPS cell-derived cardiomyocytes. J Pharmacol Toxicol Methods. 2017;84:111-27.
145. Slobbe P, Windhorst AD, Stigter-van Walsum M, Schuit RC, Smit EF, Niessen HG, et al. Development of [18F]afatinib as new TKI-PET tracer for EGFR positive tumors. Nucl Med Biol. 2014;41(9):749-57.
146. Wang X, Wang S, Lin F, Zhang Q, Chen H, Wang X, et al. Pharmacokinetics and tissue distribution model of cabozantinib in rat determined by UPLC-MS/MS. J Chromatogr B Analyt Technol Biomed Life Sci. 2015;983-984(1873-376X (Electronic)):125-31.
147. Lin Q, Zhang Y, Fu Z, Hu B, Si Z, Zhao Y, et al. Synthesis and evaluation of 18F labeled crizotinib derivative [18F]FPC as a novel PET probe for imaging c-MET-positive NSCLC tumor. Bioorg Med Chem. 2020;28(15):115577.
148. He K, Lago Mw Fau - Iyer RA, Iyer Ra Fau - Shyu W-C, Shyu Wc Fau - Humphreys WG, Humphreys Wg Fau - Christopher LJ, Christopher LJ. Lacteal secretion, fetal and maternal tissue distribution of dasatinib in rats. Drug Metab Dispos. 2008;36(12):2564-70.
149. Jain A, Kameswaran M, Pandey U, Prabhash K, Sarma HD, Dash A. 68Ga labeled Erlotinib: A novel PET probe for imaging EGFR over-expressing tumors. Bioorg Med Chem Lett. 2017;27(19):4552-7.
150. Kumar P, Singh B, Ghai A, Chuttani K, Dhawan D, Mittal BR, et al. Preclinical evaluation of (99m) Tc labeled gefitinib as a potential scintigraphic probe for the detection of tumors expressing epidermal growth factor receptors. Appl Radiat Isot. 2015;99:41-5.
151. Prause M, Niedermoser S, Wängler C, Decristoforo C, Seibold U, Riester S, et al. Synthesis, in vitro and in vivo evaluation of 18F-fluoronorimatinib as radiotracer for Imatinib-sensitive gastrointestinal stromal tumors. Nucl Med Biol. 2018;57:1-11.

152. Wan X, Zheng X, Pang X, Pang Z, Zhao J, Zhang Z, et al. Lapatinib-loaded human serum albumin nanoparticles for the prevention and treatment of triple-negative breast cancer metastasis to the brain. Oncotarget. 2016;7(23):34038-51.
153. Wang P, Peng Y, Zhang X, Fei F, Wang S, Feng S, et al. Liquid Chromatography-Mass Spectrometry/Mass Spectrometry Analysis and Pharmacokinetic Assessment of Ponatinib in Sprague-Dawley Rats. Oncol Ther. 2016;4(1):117-28.
154. Tsai TA-O, Chen YA-O, Wang LY, Hsieh CA-O. Effect of Synchronous Versus Sequential Regimens on the Pharmacokinetics and Biodistribution of Regorafenib with Irradiation. Pharmaceutics. 2021;13(3):386.
155. Yang S, Zhang B, Gong X, Wang T, Liu Y, Zhang N. In vivo biodistribution, biocompatibility, and efficacy of sorafenib-loaded lipid-based nanosuspensions evaluated experimentally in cancer. Int J Nanomedicine. 2016;11:2329-43.
156. Chen X, Wang Z Fau - Liu M, Liu M Fau - Liao M, Liao M Fau - Wang X, Wang X Fau - Du H, Du H Fau - Chen J, et al. Determination of sunitinib and its active metabolite, N-desethyl sunitinib in mouse plasma and tissues by UPLC-MS/MS: assay development and application to pharmacokinetic and tissue distribution studies. Biomed Chromatogr. 2015;29(5):679-88.
157. Pratt EC, Isaac E, Stater EP, Yang G, Ouerfelli O, Pillarsetty N, et al. Synthesis of the PET Tracer 124I-Trametinib for MAPK/ERK Kinase Distribution and Resistance Monitoring. J Nucl Med. 2020;61(12):1845-50.
158. Slobbe P, Windhorst AD, Adamzek K, Bolijn M, Schuit RC, Heideman DAM, et al. Development of [11C]vemurafenib employing a carbon-11 carbonylative Stille coupling and preliminary evaluation in mice bearing melanoma tumor xenografts. Oncotarget. 2017;8(24):38337-50.
159. Utsey K, Gastonguay MS, Russell S, Freling R, Riggs MM, Elmokadem A. Quantification of the Impact of Partition Coefficient Prediction Methods on Physiologically Based Pharmacokinetic Model Output Using a Standardized Tissue Composition. Drug Metabolism and Disposition: The Biological Fate of Chemicals. 2020;48(10):903-16.
160. Sharma AA-O, Burridge PW, McKeithan WL, Serrano RA-O, Shukla P, Sayed N, et al. High-throughput screening of tyrosine kinase inhibitor cardiotoxicity with human induced pluripotent stem cells. Sci Transl Med. 2017;9(377):eaaf2584.
161. Matsui T, Miyamoto K, Yamanaka K, Okai Y, Kaushik EP, Harada K, et al. Cell-based two-dimensional morphological assessment system to predict cancer drug-induced cardiotoxicity using human induced pluripotent stem cell-derived cardiomyocytes. Toxicol Appl Pharmacol. 2019;383:114761.
162. Sirenko O, Hancock MK, Crittenden C, Hammer M, Keating S, Carlson CB, et al. Phenotypic Assays for Characterizing Compound Effects on Induced Pluripotent Stem Cell-Derived Cardiac Spheroids. Assay Drug Dev Technol. 2017;15(6):280-96.
163. Archer CR, Sargeant R, Basak J, Pilling J, Barnes JR, Pointon A. Characterization and Validation of a Human 3D Cardiac Microtissue for the Assessment of Changes in Cardiac Pathology. Sci Rep. 2018;8(1):10160.
